# Variables influencing wearable sensor outcome estimates in individuals with stroke and incomplete spinal cord injury: a pilot investigation validating two research grade sensors

**DOI:** 10.1186/s12984-018-0358-y

**Published:** 2018-03-13

**Authors:** Chandrasekaran Jayaraman, Chaithanya Krishna Mummidisetty, Alannah Mannix-Slobig, Lori McGee Koch, Arun Jayaraman

**Affiliations:** 1 Shirley Ryan AbilityLab, Center for Bionic Medicine, Chicago, IL USA; 2 Feinberg School of Medicine, Northwestern University, Chicago, IL USA; 3 Northwestern University, Departments of Physical Medicine & Rehabilitation and Medical Social Sciences, Shirley Ryan Abilitylab, 355 E Erie St., Chicago, IL 60611 USA

**Keywords:** Wearable devices, ActiGraph, Metria-IH1, Validation, Step counts, Energy expenditure, Metabolic equivalent, Stroke, Spinal cord injury, Sweat rate

## Abstract

**Background:**

Monitoring physical activity and leveraging wearable sensor technologies to facilitate active living in individuals with neurological impairment has been shown to yield benefits in terms of health and quality of living. In this context, accurate measurement of physical activity estimates from these sensors are vital. However, wearable sensor manufacturers generally only provide standard proprietary algorithms based off of healthy individuals to estimate physical activity metrics which may lead to inaccurate estimates in population with neurological impairment like stroke and incomplete spinal cord injury (iSCI). The main objective of this cross-sectional investigation was to evaluate the validity of physical activity estimates provided by standard proprietary algorithms for individuals with stroke and iSCI. Two research grade wearable sensors used in clinical settings were chosen and the outcome metrics estimated using standard proprietary algorithms were validated against designated golden standard measures (Cosmed K4B2 for energy expenditure and metabolic equivalent and manual tallying for step counts). The influence of sensor location, sensor type and activity characteristics were also studied.

**Methods:**

28 participants (Healthy (*n* = 10); incomplete SCI (*n* = 8); stroke (n = 10)) performed a spectrum of activities in a laboratory setting using two wearable sensors (ActiGraph and Metria-IH1) at different body locations. Manufacturer provided standard proprietary algorithms estimated the step count, energy expenditure (EE) and metabolic equivalent (MET). These estimates were compared with the estimates from gold standard measures. For verifying validity, a series of Kruskal Wallis ANOVA tests (Games-Howell multiple comparison for post-hoc analyses) were conducted to compare the mean rank and absolute agreement of outcome metrics estimated by each of the devices in comparison with the designated gold standard measurements.

**Results:**

The sensor type, sensor location, activity characteristics and the population specific condition influences the validity of estimation of physical activity metrics using standard proprietary algorithms.

**Conclusions:**

Implementing population specific customized algorithms accounting for the influences of sensor location, type and activity characteristics for estimating physical activity metrics in individuals with stroke and iSCI could be beneficial.

**Electronic supplementary material:**

The online version of this article (10.1186/s12984-018-0358-y) contains supplementary material, which is available to authorized users.

## Background

Ubiquitous estimation of physical activity and quality of life measures collected from real life environments are becoming an imperative component to monitor successful translation of clinical research into patient’s own living environment [1–3]. With context to this, continuous monitoring paradigms are gaining substantial significance in tracking a patient’s compliance to a stipulated exercise regime and to gauge their level of community integration post rehabilitation [1, 2, 4]. Commonly used methods of measuring mobility include the traditional methods of performance-based or patient-reported measures. These measurements are either limited by rater or recall bias, or in their ability to encompass all aspects of community mobility. Advanced methods like camera based motion capture, pressure sensor walkway [5] and force plate systems for assessments, although significantly reliable, limits data collection to a confined controlled laboratory space and are expensive [6]. While such controlled environment tests can provide high-resolution information to uncover the underlying biomechanics during in-patient movement assessments, they provide very little to no information about a patient's natural physical activity behavior and compliance in their community or home setting [7].

Evidence suggests that employing wearable sensors provide means to remotely and continuously track patient’s recovery in real world settings [7, 8]. Indeed, activity monitoring with wearables are showing cost benefits for healthcare and also paving the way for participatory clinical decision making for customized healthcare [1, 2, 9–11]. Despite offering numerous benefits, the validity and reliability of outcome estimates from these wearable sensors for rehabilitation medicine in individuals with chronic conditions is a daunting challenge for researchers [12, 13].

A potential reason for this being that most of standard proprietary algorithm (SPA) provided by sensor manufacturer’s are derived using empirical data from healthy populations leading to inaccurate/unreliable estimates when deployed to estimate outcome measures in clinical populations [13]. Research to date acknowledges two major contributing factors to such estimation inaccuracies [14], namely, (i) the sensor location and (ii) variation in acceleration thresholds due to pathology specific movement signatures in comparison to healthy controls.

In this context, there has been less focus on studying the influence of such factors on outcome metrics other than step count, like, energy expenditure (EE) and metabolic equivalents (MET), especially in individuals with stroke and incomplete spinal cord injury (iSCI) [13, 15, 16]. Further, there is limited information regarding the relationship between attributes like (i) sensor type (i.e. body worn, body stuck and standalone Vs fusion sensor modalities) and (ii) characteristics of activity being studied, on the validity of outcome measures estimated using SPAs in populations with neurological impairments like iSCI and stroke.

Consequently, our primary goal was to investigate the influence of sensor type, and sensor location on the validity of physical activity outcome estimates (step count, energy expenditure (EE) and metabolic equivalent (MET) as provided by SPA from wearable sensors in a sample of healthy individuals (controls) and individuals with iSCI (ambulatory) and stroke. A secondary goal was to investigate the influence of activity characteristics (intensity) on the validity of each of the physical activity outcome estimates in our sample. In this pilot study, we investigated the influence of above aspects on validity of the outcome measures as provided by the respective SPAs from two research grade sensors. The spectrum of activities studied were identical to those encountered in activities of daily living (ADL), but were performed in a controlled laboratory setting. Although performed in a controlled environment, such findings will have important implications for understanding the possible factors that needs to be considered while estimating outcome measures using wearables in free living conditions.

It was postulated that the choice of sensor type, sensor location (arm, waist and ankle), population specific movement signatures (healthy, iSCI (ambulatory), stroke) and the characteristics of the activity being studied will significantly influence the validity of the physical activity outcomes metrics estimated using SPA in laboratory conditions.

## Methods

The participant pool included, healthy controls, and individuals with iSCI and chronic stroke who could ambulate with or without an assistive device. Detailed group wise demographic information is provided in Table 1. Exclusion criteria included, (i) presence of any known serious cardiac conditions, (ii) neurological degenerative pathologies as co-morbidities (such as Multiple Sclerosis, Alzheimer’s disease, Parkinson’s disease, etc.), and (iii) inability to sit unsupported. In addition, subjects were requested to stay off of any medications that has previously known to affect their metabolism during the study period.

**Table 1:** Sample demographics and self-reported perceived Borg RPE

**Characteristics**	**Healthy**	**iSCI**	**Stroke**
Age (Yrs)	27.1 (5.1)	48.5 (10.4)	55.6 (9.4)
Height (cm)	173.7 (8.3)	179.1 (8.4)	172.1 (8.5)
Weight (lbs)	155.5 (33.6)	186.9 (37.8)	190.1 (33.9)
Gender (M/F)	M (n = 6); F (*n* = 4)	M (*n* = 7); F (*n* = 1)	M (n = 6); F (n = 4)
Impairment demographics	–	C3-C4 (n = 1); C5-C6 (n = 1); C1-C4 (n = 1); C4 (n = 1); C6-C7 (n = 1); C7 (n = 1); T8-T9 (n = 1); L3-L4 (n = 1).	Right side impaired (n = 4); Left side impaired (*n* = 6). Hemorrhagic (n = 4); Ischemic (n = 6).
Time since condition (Years)	–	11.9 (7.7)	7.0 (5.0)
Assistive devices used during testing	–	Walker and knee brace (n = 1)	Straight cane (*n* = 2)
**BORG RPE for activities**			
Lying	7(1)	8 (5)	6 (0)
Sitting	6 (0)	8 (3)	7 (1)
Standing	6 (0)	9 (5)	9 (4)
50 step walking	7 (1)	10 (4)	8 (3)
6MWT	10 (2)	15 (3)	14 (3)
Multi sit-to-stand	12 (2)	14 (4)	15 (3)

### Devices used

**Fig. 1:**
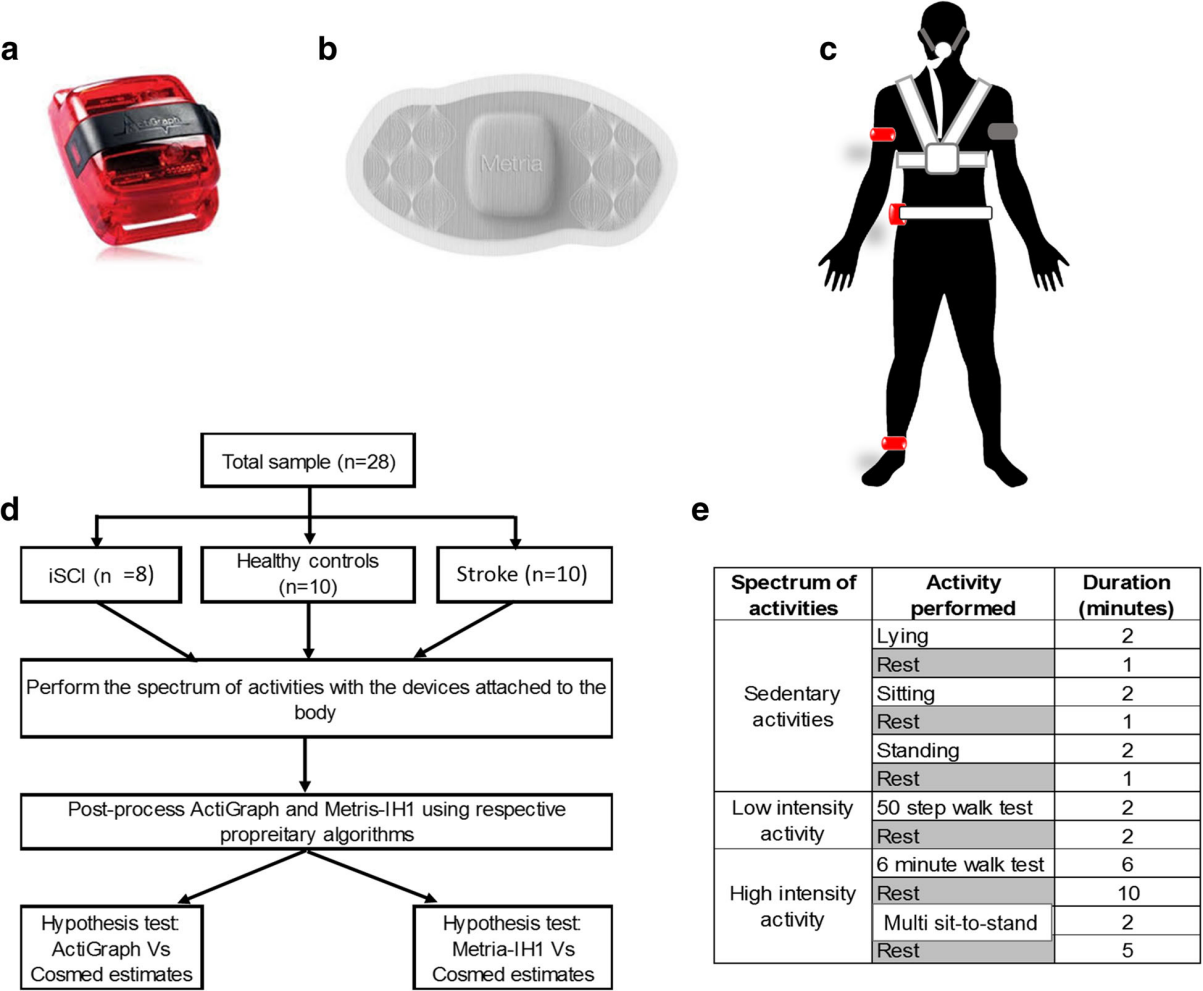
Devices used and protocol design. **a** Picture of ActiGraph wG3TX-BT; **b** picture of Metria-IH1; **c** The sensor locations used, ActiGraphs (red color) were placed on the right side upper arm, waist and ankle while the Metria-IH1 (grey color) was placed on the back side of left upper arm. The Cosmed K4B2 was body mounted with the rubberized facemask; **d**) the experimental design and the spectrum of activities executed during the protocol. To execute the study protocol, participants performed a set of structured indoor activities in a controlled laboratory setting. (**e**) The spectrum of the performed activities was categorized into three levels, (i) sedentary activities: lying down on a treatment table, sitting and standing (with or without assistive device) for two minutes each, (ii) low intensity activity: walk 50 steps, and (iii) high intensity activities: a six-minute walking test (6MWT) and two minute of fast paced multi sit-to-stand activity. Sufficient rests and recovery were provided between all the performed activities. All the three devices, namely, the Actigrpah, Metria and Cosmed K4B2 continuously collected data during the entire protocol

Currently, a plethora of commercial and research grade wearable devices are available for clinicians to choose from [17]. Testing the validity of outcome metrics from all the devices was outside the scope of this study design. Therefore, in order to test the postulated hypotheses two specific research grade wearable sensor types used in clinical research, namely, Actigraphs [18, 19], and Metria-IH1 [18, 20] were chosen (Fig. 1a & 1b).

The goal of the study was to investigate the validity of estimates as provided by the respective SPAs from ActiLife and SenseWear. These SPAs are usually based on data from healthy individuals and the algorithms performance are optimized to the specific sensor locations. Therefore, the sensor locations for this study were chosen based on literature and the respective manufacturers prescription [21]. This procedure was adopted to reduce any possible confounding factors to the outcome estimates that may arise due to switching of the sensor location from the optimal location for which the respective SPAs were developed. Furthermore, all wearable devices used in this study were obtained from the same manufacturing batch. This was done to minimize any measurement differences inherently arising due to variations in manufacturing process.

#### ActiGraph

The ActiGraph wGT3X-BT’s [22] were worn on the upper arm [23], the waist [24, 25] and the ankle [26] (Fig. 1c). The waist sensor locations were chosen  based on previous literature [13, 24]. For consistency, all the ActiGraphs were placed on the right side of the body. Adjustable fabric belts securely positioned the ActiGraphs on their respective locations. ActiGraphs measure the triaxial acceleration to estimate physical activity metrics [22]. (Fig. 1a). The ActiGraphs sampled at 30 Hz.

Each ActiGraph device was assigned to a specific anatomical location. The device to anatomical location was held consistent between participants to minimize confounding factors due to unit calibration and sensor switching [27]. The time on all the three devices were synchronized to the local atomic clock server time before  data collection began.

#### Metria-IH1

Based on the manufacturer’s recommendation, the Metria-IH1 patch was adhered to the skin and located on the back of the upper left arm (Fig. 1b, c). The Metria IH1 patch houses variety of sensors to measure four modalities, namely, (i) 3-axis accelerometer, (ii) skin temperature, (iii) near body temperature and (iv) galvanic skin response (GSR). The module sampled data at the rate of 5000 data points per minute. The accelerometer alone sampled at 32 Hz. Metria-IH1 is a one-time use and throw device.

#### Outcome measures

Three outcome metrics of relevance widely used in monitoring physical activity in clinical rehabilitation in individuals with stroke and iSCI were studied, namely, (i) step count, (ii) energy expenditure (EE) in Kcals, and (ii) metabolic energy (METs) [14, 28, 29]. These metrics were compared against the designated gold standard (Cosmed K4B2 [30] for EE and MET and phone-based counter for step count).

#### Gold standard devices

Cosmed K4B2 (K4B2 Cosmed, Italy) [30] is a portable gas analysis system that measures oxygen consumption and Carbon-dioxide production in a breath by breath fashion. Extensive scientific usage and validation of Cosmed K4B2 makes it a gold standard in breath-by-breath metabolic measurements [22, 31]. For EE and MET metrics, the Cosmed K4B2 output was used as the gold standard comparison.

For step counts, the steps taken were counted manually using a phone-based counter during the 50 step walk test. This manual tally was used as gold standard for step count [32, 33].

### Experimental data collection

The experimental design and the test protocol are presented in Fig. 1(d) & (e). Consistent with recommended practice, the validation protocol was designed to cover a spectrum of physical activities [27]. Data collection started with the subjects performing a series of activities such as lying, sitting, and standing for two-minutes each, a 50 steps walk on a hallway [34], a six minute walking test (6MWT) and finally two minutes of multi sit-to-stands. During multi sit-to-stand task, subjects were encouraged to do as many sit-to-stand sets as they safely and possibly can do.

Sufficient rests periods were given to the participants between each activity. The rest duration between activities were to ensure that the heart rate of the participants returned to their resting levels before starting the proceeding activity (refer Fig. 1(e)).

At the end of each activity, subjects self-reported their perceived effort of each activity using a Borg scale of perceived exertion [35]. These self-reported ratings were later used to classify the intensity of activities performed based on the exertion levels. Participants were given the choice on whether they wanted to use their assistive device. All the participant’s completed the entire protocol successfully in a single visit. All the three devices, namely, the ActiGraphs, Metria and Cosmed K4B^2^ continuously and simultaneously recorded data during the entire session (i.e. 60 min).

### Data analysis

For all data analysis, SPA provided by manufacturers of the respective devices were used for estimating the outcome metrics of interest (EE, MET and step count).

*ActiLife:*. The goal was to study the validity of outcome metrics while using SPAs in individuals with stroke and iSCI. The Choi [36] and Freedson [37] proprietary algorithms based off of healthy population empirical data was used to extract the EE and MET estimates for each of the activities in all of the groups. A Harris Benedict equation [38] was used to include the contribution of BMR to estimated EE and MET’s. This data extraction procedure was repeated for each of the wearable sensors positioned at the waist, ankle and the arm. The metrics from ActiGraph’s were used to study the effect of the sensor location, activity characteristics and population effects on the validity of outcome estimates.

*Metria-IH1:* The Metria-IH1’s proprietary fusion algorithm developed on the SenseWear software development kit platform, prompts for anthropometric information (age, height, weight, gender, dominant hand and smoking) at the time of data processing. This information is then fused along with information from the various on board sensor modules to calculate the outcome metrics of interest [20, 39]. The outcome metrics from Metria-IH1 were used to study the effect of activity characteristics and population effects on the validity of outcome estimates.

*COSMED-K4B2* [30]**:** The manufacturer provided software was used to extract the EE and MET estimates from the Cosmed K4B2 [30, 39].

*Step count*: For agreement, the manually counted steps using a phone based tally counter was cross-verified with step counts from the video recorded during the 50 step walk test [32, 33].

### Statistical analysis

All the statistical analyses were performed using IBM SPSS 21.0 (SPSS Inc., Chicago, IL). Per study design, there were no direct between group comparisons (i.e. no direct comparison between healthy, iSCi and Stroke) and between device comparisons (i.e. no direct comparison between ActiGraph Vs Metria-IH1). For each sensor the postulated hypotheses were statistically compared with the designated gold standard’s estimate (i.e. EE & MET: Device vs. Cosmed K4B2; step count: device count vs. manual tally). The null hypothesis (H0) was that, the mean ranks of the groups (device estimates Vs Cosmed estimates) are the same. Therefore, failure to achieve the statistical significance, does not give sufficient evidence to reject the H0.

The statistical significance was set to *p* < .05 for all hypotheses tests pertaining to Metria-IH1. To account for multiple comparisons a corrected (Bonferroni) *p* value of *p* < .016 was used for all hypothesis testing pertaining to ActiLife estimates. For the group with stroke, data were analyzed based on the side of stroke impairment. (i.e. (Metria-IH1 for stroke (L) and ActiGraphs for stroke (R))).

Following epsilon-squared effect size (E^2^) thresholds were used for interpretation of strength of relationship: small effect size (E^2^ ≤ .1), medium effect size (.1 < E^2^ ≤ .3) and large effect size (E^2^ > .5) [40]. With context to this analysis the following interpretation was used: a value of E^2^ = 1.0, indicated a large deviation and a value of E^2^ = 0 indicated a closer match in the mean rank of the estimated outcome with the designated golden standards (Cosmed K4B2 for EE and MET, manual step tally for step count).

Due to the smaller sample size and non-normal distribution of the data (based on Shapiro-Wilk test), non-parametric tests were used to statistically verify the postulated hypotheses. A series of Kruskal Wallis tests (Games-Howell multiple comparison for post-hoc analyses due to unequal variances verified using Levene’s test of equality for variances) were conducted to compare the mean rank and absolute agreement of outcome metrics estimated by each of the devices in comparison with Cosmed (designated gold-standard measurements [30]. To verify step count validity, estimates of step count from ActiLife and Metria-IH1 SPAs were statistically compared with the manually counted steps during the 50 steps walk test.

## Results

For brevity only salient results are reported in the text. For detailed statistical results on bias between measures please refer to the Tables 1, 2 & 3. For detailed analysis of absolute agreement to assess reliability refer to the post-hoc analysis (additional file shows this in more detail [see Additional file 1; Supplementary Tables ST1 through ST8]). The results for the outcome metrics trended the same way from both the bias (Kruskal-Wallis) and absolute agreement tests (Games-Howell post-hoc).

**Table 2:** Energy expenditure (EE) estimates for different group using SPA

	*EE estimates from standard algorithm.* *Mean [95% CI [LB, UB] in kCals / min*					Kruskal Wallis test statistics	Effect size
Activity/Group	A arm (right)	A waist (right)	A ankle (right)	Metria (M arm (left))	Cosmed K4B2	χ2, p, mean rank [device, cosmed]	E^2^ = $\left\{\frac{x2}{\frac{\left({N}^2-1\right)}{\left(N+1\right)}}\right\}$
Lying							
Healthy	0.07 [−.04, .17]*	0.03 [−.01, .06]*	0.06 [−.01, .13]*	2.04 [1.82, 2.26] †	3.2 [2.67, 3.72]	A arm: 14.3, *p* < .001, [5.5, 15.5] A waist: 14.3, *p* < .001, [5.5, 15.5] A ankle: 14.3, p < .001, [5.5, 15.5] M arm: 13.7, p < .001, [5.6, 15.4]	A arm: .75 A waist: .75 A ankle: .75 M arm: .72
iSCI	.001 [.001, .001]*	.001 [.001, .001]*	.001 [.001, .001]*	1.86 [1.42, 2.31]	1.59 [1.26, 1.91]	A arm: 11.3, *p* = .001, [4.5, 12.5] A waist: 11.3, p = .001, [4.5, 12.5] A ankle: 11.3, p = .001, [4.5, 12.5] M arm: 1.1, *p* = .3, [9.7,7.2]	A arm: .75 A waist: .75 A ankle: .75 M arm: .07
Stroke (L)	.002 [.00, .006]*	.118 [−.183, .421]*	.02 [−.03, .015]*	1.65 [1.30,1.99]	1.59 [1.18, 1.99]	A arm: 8.3, *p* = .004, [3.5, 9.5] A waist: 8.3, p = .004, [3.5, 9.5] A ankle: 8.3, p = .004, [3.5, 9.5]M arm: 0.4, *p* = .5, [7.1, 5.8]	A arm: .76 A waist: .76 ankle: .76 M arm: .04
Stroke (R)	.001 [.001, .001]*	.001 [.001, .001]*	.001 [.001, .001]*	1.59 [1.13, 2.05]	1.65 [.99, 2.30]	A arm: 5.3, *p* = .02, [2.5, 6.5] A waist: 5.3, *p* = .02, [2.5, 6.5] A ankle: 5.3, p = .02, [2.5, 6.5] M arm: 0.0, *p* = .99, [4.5, 4.5]	A arm: .76 A waist: .76 A ankle: .76 M arm: .00
Sitting							
Healthy	.031 [−.003, .070]*	.003 [.001, .005]*	.009 [−.006, .024]*	2.33 [1.94, 2.73]	2.08 [1.82, 2.35]	A arm: 14.3, *p* < .001, [5.5, 15.5] A waist: 14.3, p < .001, [5.5, 15.5] A ankle: 14.3, p < .001, [5.5, 15.5] M arm: 1.3, *p* = .3, [12.0,9.0]	A arm: .75 A waist: .75 A ankle: .75 M arm: .07
iSCI	.04 [−.025, .120]*	.003 [−.001, .008]*	.001 [.001, .001]*	1.92 [1.47, 2.37]	1.48 [1.25, 1.71]	A arm: 11.3, *p* = .001, [4.5, 12.5] A waist: 11.3, p = .001, [4.5, 12.5] A ankle: 11.3, p = .001, [4.5, 12.5] M arm: 2.8, *p* = .1, [10.5,6.5]	A arm: .75 A waist: .75 A ankle: .75 M arm: .20
Stroke (L)	.05 [−.054, .152]*	.050 [−.065, .166]*	.017 [−.015, .05]*	1.64 [1.32,1.95]	1.31 [1.04, 1.57]	A arm: 8.3, *p* = .004, [3.5, 9.5] A waist: 8.3, p = .004, [3.5, 9.5] A ankle: 8.3, p = .004, [3.5, 9.5] M arm: 2.8, p = .1, [8.25,4.75]	A arm: .76 A waist: .76 A ankle: .76 M arm: .26
Stroke (R)	.001 [.001, .001]*	.001 [.001, .001]*	.001 [.001, .001]*	1.58 [1.16, 1.99] †	1.19 [.96, 1.43]	A arm: 5.3, *p* = .02, [2.5, 6.5] A waist: 5.3, p = .02, [2.5, 6.5] A ankle: 5.3, p = .02, [2.5, 6.5] M arm: 4.1, *p* = .04, [6.2, 2.7]	A arm: .76 A waist: .76 A ankle: .76 M arm: .58
Standing							
Healthy	.015 [−.007, .038]*	.002 [.002, .003]*	.004 [.00, .008]*	2.11 [1.68, 2.52]	1.94 [1.65, 2.23]	A arm: 14.3, p < .001, [5.5, 15.5] A waist: 14.3, p < .001, [5.5, 15.5] A ankle: 14.3, p < .001, [5.5, 15.5] M arm: .28, *p* = .6, [11.2, 9.8]	A arm: .75 A waist: .75 A ankle: .75 M arm: .01
iSCI	.022 [−.026, .072]*	.001 [.001, .001]*	.001 [.001, .001]*	2.17 [1.32, 3.02]	1.74 [1.34, 2.13]	A arm: 11.3, p = .001, [4.5, 12.5] A waist: 11.3, p = .001, [4.5, 12.5] A ankle: 11.3, p = .001, [4.5, 12.5] M arm: .54, *p* = .5, [9.3,7.6]	A arm: .75 A waist: .75 A ankle: .75 M arm: .04
Stroke (L)	1.53 [−.24, .55]*	.002 [.00, .003]*	.011 [−.015, .037]*	1.70 [1.33,2.08]	1.55 [1.11,1.97]	A arm: 8.3, p = .004, [3.5, 9.5] A waist: 8.3, p = .004, [3.5, 9.5] A ankle: 8.3, p = .004, [3.5, 9.5] M arm: .9, *p* = .3, [7.5, 5.5]	A arm: .76 A waist: .76 A ankle: .76 M arm: .08
Stroke (R)	.001 [.001, .001]*	.001 [.001, .001]*	.001 [.001, .001]*	1.6 [1.17, 2.02]	1.42 [.81, 2.04]	A arm: 5.3, *p* = .02, [2.5, 6.5] A waist: 5.3, p = .02, [2.5, 6.5] A ankle: 5.3, p = .02, [2.5, 6.5] M arm: .7, *p* = .4, [5.2, 3.7]	A arm: .76 A waist: .76 A ankle: .76 M arm: .11
50 steps walk							
Healthy	2.02 [1.54, 2.50]*	1.78 [1.29, 2.26]*	4.21 [3.38, 5.03]	2.16 [1.89, 2.44] †	2.98 [2.44, 3.52]	A arm: 7.0, *p* = .008, [7.0, 14.0] A waist: 10.1, *p* = .001, [6.3, 14.7] A ankle: 5.5, p = .02, [13.6, 7.4] M arm: 7.0, p = .008, [7.0,14.0]	A arm: .37 A waist: .53 A ankle: .29 M arm: .37
iSCI	1.26 [.65, 1.88]*	.64 [.18, 1.09]*	2.71 [.35, 5.07]	2.71 [.79, 4.63] †	3.52 [2.47, 4.57]	A arm: 9.3, *p* = .002, [4.8, 12.3] A waist: 11.3, p = .001, [4.5, 12.5] A ankle: 2.2, *p* = .14, [6.7, 10.2] M arm: 5.8, p = .02, [5.6,11.4]	A arm: .62 A waist: .75 A ankle: .14 M arm: .39
Stroke (L)	2.65 [.63, 4.67]	1.67 [−.58, 3.93]	5.75 [3.38, 8.12]*	1.77 [1.26,2.29] †	2.78 [1.92, 3.65]	A arm: .6, *p* = .4, [5.7, 7.3] A waist: 1.3, p = .3, [5.3, 7.6] A ankle: 6.6, *p* = .01, [9.1, 3.8] M arm: 6.5, p = .01, [3.8, 9.1]	A arm: .06 A waist: .11 A ankle: .60 M arm: .41
Stroke (R)	2.83 [.67, 4.98]	2.0 [.93, 3.06]	2.97 [.04, 5.90]	1.91 [.95, 2.86]	2.74 [1.72, 3.76]	A arm: .1, *p* = .8, [4.2, 4.7] A waist: .7, p = .4, [3.7, 5.2] A ankle: .1, *p* = .8, [4.2, 4.7] M arm: 2.1, *p* = .15, [3.2, 5.7]	A arm: .01 A waist: .11 A ankle: .01 M arm: .30
6 min walk test							
Healthy	5.9 [3.85, 7.95]	6.22 [4.23, 8.20]	9.73 [8.36, 11.1]*	5.98 [5.21, 6.75]	6.63 [5.78, 7.48]	A arm: .3, p = .6, [9.8, 11.2] A waist: .02, *p* = .9, [10.3, 10.7] A ankle: 11.1, *p* = .001, [14.9, 6.1] M arm: 1.0, p = .3, [9.2, 11.8]	A arm: .01 A waist: .00 A ankle: .58 M arm: .05
iSCI	2.07 [.46, 3.68]*	.78 [.13,1.43]*	2.05 [.46, 3.65]*	5.32 [3.11, 7.54]	4.94 [3.3, 6.57]	A arm: 6.4, *p* = .012, [5.5, 11.5] A waist: 11.3, *p* = 0.001, [4.5, 12.5] A ankle: 6.3, p = .012, [5.5, 11.5] M arm: .04, p = .8, [8.7,8.2]	A arm: .42 A waist: .75 A ankle: .42 M arm: .00
Stroke (L)	6.11 [1.42, 10.81]	4.69 [−1.91, 10.58]	9.54 [4.97, 14.11]	5.37 [2.65,8.10]	5.81 [1.97,9.66]	A arm: .03, *p* = .9, [6.3, 6.7] A waist: 1.3, p = .3, [5.3, 7.7] A ankle: 4.3, *p* = .04, [8.7, 4.3] M arm: .03, p = .9, [6.3, 6.7]	A arm: .00 A waist: .11 A ankle: .39 M arm: .00
Stroke (R)	6.96 [4.16, 9.76]	6.10 [4.03, 8.17]	6.76 [4.01, 9.52]	4.56 [3.35, 5.77]	6.27 [4.17, 8.36]	A arm: .1, p = .8, [4.7, 4.2] A waist: .1, p = .8, [4.7, 4.2] A ankle: .1, p = .8, [4.7, 4.2] M arm: 3, *p* = .08, [3.0, 6.0]	A arm: .01 A waist: .01 A ankle: .01 M arm: .43
Multi-sit-to-stand							
Healthy	7.92 [5.91, 9.93]	6.02 [3.91, 8.13]	.151 [−.160, .462]*	3.55 [2.94, 4.16] †	6.79 [5.00, 8.59]	A arm: .7, *p* = .4, [11.6, 9.4] A waist: .8, p = .4, [9.3, 11.7] A ankle: 14.3, p < .001, [5.5,15.5] M arm: 9.1, *p* = .002, [6.5, 14.5]	A arm: .04 A waist: .04 A ankle: .75 M arm: .48
iSCI	4.64 [2.4, 6.9]	1.53 [.66, 2.39]*	.05 [−.011, .104]*	3.33 [1.35, 5.31]	3.82 [3.19, 4.44]	A arm: .5, p = .5, [9.4, 7.6] A waist: 9.9, p = .002, [4.7, 12.2] A ankle: 11.3, *p* = .001, [4.5, 12.5] M arm: 2.8, *p* = .1, [6.5,10.5]	A arm: .04 A waist: .66 A ankle: .75 M arm: .19
Stroke (L)	6.56 [3.2, 9.92]	5.45 [1.53, 9.38]	.11 [.00, .22]*	2.03 [1.58,2.48] †	4.95 [2.64, 7.26]	A arm: .9, *p* = .3, [7.5, 5.5] A waist: .00, *p* = .99, [6.5, 6.5] A ankle: 8.3, *p* = .004, [3.5, 9.5] M arm: 7.4, *p* = .006, [3.7, 9.3]	A arm: .08 A waist: .00 A ankle: .76 M arm: .67
Stroke (R)	2.89 [.46, 5.33]	5.37 [2.52, 8.21]	.22 [−.36, .80]	2.1 [1.27, 2.93] †	4.75 [3.32, 6.19]	A arm: 3, *p* = .08, [3.0,6.0] A waist: .3, *p* = .6, [5.0, 4.0] A ankle: 5.3, *p* = .02, [2.5, 6.5] M arm: 5.3, p = .02, [2.5, 6.5]	A arm: .43 A waist: .05 A ankle: .76 M arm: .76

†p < 0.05 (Metria-IH1); *p < 0.016 (ActiGraph)

**Table 3:** Metabolic equivalent (MET) estimates for different group using SPA

Activity/Group	* MET estimates from standard algorithm.* *Mean [95% CI [LB, UB]*					Kruskal Wallis test statistics	Effect size
	A arm (right)	A waist (right)	A ankle (right)	Metria (M arm (left))	Cosmed K4B2	χ2, p, mean rank [device, cosmed]	E^2^ = $\left\{\frac{x^2}{\frac{\left({N}^2-1\right)}{\left(N+1\right)}}\right\}$
Lying							
Healthy	1.03 [0.97, 1.08]*	1.02 [0.99, 1.00]*	1.03 [1.00, 1.05]*	1.79 [1.52, 2.05] †	2.33 [1.86, 2.79]	A arm: 9.7, p = .002, [6.5, 14.5] A waist: 9.7, p = .002, [6.5, 14.5] A ankle: 9.7, p = .002, [6.5, 14.5] M arm: 5.4, *p* = .02, [7.5, 13.5]	A arm: .51 A waist: .51 A ankle: .51 M arm: .28
iSCI	1.00 [.001, .001]*	1.00 [.001, .001]*	1.00 [.001, .001]*	1.19 [1.06, 1.31]	1.12 [0.94, 1.30]	A arm: 3.2, *p* = .07, [6.5, 10.5] A waist: 3.2, p = .07, [6.5, 10.5] A ankle: 3.2, p = .07, [6.5, 10.5] M arm: .71, *p* = .4, [9.5,7.5]	A arm: .21 A waist: .21 A ankle: .21 M arm: .05
Stroke (L)	1.00 [.001,.001]	1.01 [0.97, 1.07]	1.00 [.001,.001]	1.13 [0.94, 1.33]	1.07 [1.01, 1.13]	A arm: 4.2, *p* = .04, [4.5, 8.5] A waist: 1.8, *p* = .2, [5.1, 7.8] A ankle: 4.2, p = .04, [4.5, 8.5] M arm: 0.03, *p* = .9, [6.7, 6.3]	A arm: .38 A waist: .16 A ankle: .38 M arm: .00
Stroke (R)	1.00 [.001, .001]*	1.00 [.001, .001]*	1.00 [.001, .001]*	1.14 [1.02, 1.27]	1.16 [.76, 1.57]	A arm: 1.5, p = .2, [3.5, 5.5] A waist: 1.5, p = .2, [3.5, 5.5] A ankle: 1.5, p = .2, [3.5, 5.5] M arm: 0.1, *p* = .8, [4.2, 4.7]	A arm: .21 A waist: .21 A ankle: .21 M arm: .01
Sitting							
Healthy	1.00 [1.00, 1.01]*	1.00 [1.00, 1.01]*	1.00 [1.00, 1.01]*	2.03 [1.66, 2.38]	1.60 [1.28, 1.92]	A arm: 10.0, *p* = .002, [6.5, 14.5] A waist: 10.4, *p* = .001, [6.5, 14.5] A ankle: 10.4, p = .001, [6.5, 14.5] M arm: 3.6, *p* = .06, [13.0,8.0]	A arm: .53 A waist: .55 A ankle: .55 M arm: .19
iSCI	1.00 [1.00, 1.01]	1.00 [1.00, 1.01]	1.00 [1.00, 1.01]	1.22 [1.10, 1.34]	1.05 [0.88, 1.21]	A arm: .8, p = .4, [7.5, 9.5] A waist: .8, p = .4, [7.5, 9.5] A ankle: .8, p = .4, [7.5, 9.5] M arm: 3.6, p = .06, [10.7,6.2]	A arm: .05 A waist: .05 A ankle: .05 M arm: .24
Stroke (L)	1.00 [.99, 1.02]	1.00 [.001, .001]	1.00 [.001, .001]	1.12 [.96, 1.29]†	.89 [0.76, 1.01]	A arm: 4.0, *p* = .05, [8.5, 4.5] A waist: 4.2, p = .04, [8.5, 4.5] A ankle: 4.2, p = .04, [8.5, 4.5] M arm: 5.8, *p* = .02, [9.0,4.0]	A arm: .36 A waist: .38 A ankle: .38 M arm: .53
Stroke (R)	1.00 [.001, .001]	1.00 [.001, .001]	1.00 [.001, .001]	1.13 [1.04, 1.22]†	.89 [0.65, 1.07]	A arm: 1.5, p = .2, [5.5, 3.5] A waist: 1.5, p = .2, [5.5, 3.5] A ankle: 1.5, p = .2, [5.5, 3.5] M arm: 5.4, p = .02, [6.5, 2.5]	A arm: .21 A waist: .21 A ankle: .21 M arm: .77
Standing							
Healthy	1.00 [.001,.001]*	1.00 [.001,.001]*	1.00 [.001,.001]*	1.81 [1.53, 2.08]	1.51 [1.23, 1.79]	A arm: 10.4, p = .001, [6.5, 14.5] A waist: 10.4, p = .001, [6.5, 14.5] A ankle: 10.4, p = .001, [6.5, 14.5] M arm: 1.5, p = .2, [12.1, 8.9]	A arm: .51 A waist: .51 A ankle: .51 M arm: .28
iSCI	1.00 [.001,.001]	1.00 [.001,.001]	1.00 [.001,.001]	1.37 [1.0, 1.75]	1.22 [1.04, 1.39]	A arm: 3.2, p = .07, [6.5, 10.5] A waist: 3.2, p = .07, [6.5, 10.5] A ankle: 3.2, p = .07, [6.5, 10.5] M arm: .01, *p* = .9, [8.6, 8.4]	A arm: .21 A waist: .21 A ankle: .21 M arm: .00
Stroke (L)	1.06 [0.90, 1.23]	1.00 [.001,.001]	1.00 [.001,.001]	1.15 [1.11, 1.21]	1.04 [0.81, 1.27]	A arm: .2, p = .6, [7.0, 6.0] A waist: 0.0, *p* = .99, [6.5, 6.5] A ankle: 0.0, p = .99, [6.5, 6.5] M arm: .6, p = .4, [7.3, 5.6]	A arm: .02 A waist: .00 A ankle: .00 M arm: .05
Stroke (R)	1.00 [.001,.001]	1.00 [.001,.001]	1.00 [.001,.001]	1.15 [1.04, 1.26]	1.01 [0.70, 1.32]	A arm: 1.5, p = .2, [5.5, 3.5] A waist: 1.5, p = .2, [5.5, 3.5] A ankle: 1.5, p = .2, [5.5, 3.5] M arm: 1.3, p = .2, [5.5, 3.5]	A arm: .21 A waist: .21 A ankle: .21 M arm: .19
50 steps walk							
Healthy	2.18 [1.73, 2.63]	2.04 [1.57, 2.51]	3.90 [3.66, 4.14]*	1.86 [1.71, 2.02]	2.41 [1.76, 3.05]	A arm: .3, p = .6, [9.8, 11.2] A waist: 1.0, *p* = .3, [9.2, 11.8] A ankle: 11.1, p = .001, [14.9, 6.1] M arm: 2.8, *p* = .1, [7.8,13.2]	A arm: .02 A waist: .05 A ankle: .58 M arm: .15
iSCI	1.19 [0.92, 1.46]*	1.01 [0.99, 1.03]*	2.08 [0.89, 3.27]	1.70 [0.75, 2.65] †	2.42 [2.01, 2.83]	A arm: 10.6, p = .001, [4.6, 12.4] A waist: 11.6, p = .001, [4.5, 12.5] A ankle: .7, p = .4, [7.5, 9.5] M arm: 5.8, p = .02, [5.6,11.4]	A arm: .71 A waist: .77 A ankle: .05 M arm: .39
Stroke (L)	1.89 [0.94, 2.84]	1.58 [0.63, 2.52]	4.04 [3.35, 4.74]*	1.20 [1.01, 1.39] †	1.85 [1.59, 2.12]	A arm: .1, *p* = .7, [6.1, 6.8] A waist: .9, p = .3, [5.5, 6.5] A ankle: 8.3, *p* = .004, [9.5, 3.5] M arm: 8.3, p = .004, [3.5, 9.5]	A arm: .01 A waist: .08 A ankle: .75 M arm: .75
Stroke (R)	2.30 [1.15, 3.45]	1.78 [1.23, 2.34]	2.43 [0.75, 4.11]	1.36 [0.88, 1.84]	1.96 [1.21, 2.71]	A arm: .7, p = .4, [5.2, 3.7] A waist: .3, p = .6, [4.0, 5.0] A ankle: .3, p = .6, [4.0, 5.0] M arm: 3.0, *p* = .08, [3.0, 6.0]	A arm: .14 A waist: .04 A ankle: .04 M arm: .43
6 min walk test							
Healthy	5.40 [4.13, 6.67]	5.55 [4.76, 6.34]	8.05 [7.25, 8.84]*	5.23 [4.47, 5.99]	5.39 [4.09, 6.70]	A arm: 0.00, p = .99, [10.5, 10.5] A waist: .1, p = .7, [11.0, 10.0] A ankle: 8.7, *p* = .003, [14.4, 6.6] M arm: .1, p = .7, [11.0, 10.0]	A arm: .00 A waist: .01 A ankle: .46 M arm: .01
iSCI	1.86 [0.92, 2.79]	1.15 [0.94, 1.37]*	2.77 [1.36, 4.18]	3.35 [2.01, 4.69]	3.34 [2.53, 4.13]	A arm: 5.3, p = .02, [5.7, 11.2] A waist: 11.5, *p* = 0.001, [4.5, 12.5] A ankle: .5, *p* = .5, [7.6, 9.4] M arm: .1, p = .7, [8.1, 8.9]	A arm: .35 A waist: .77 A ankle: .03 M arm: .01
Stroke (L)	3.93 [1.75, 6.11]	2.87 [0.13, 5.74]	6.27 [4.81, 7.74]	3.54 [2.53, 4.56]	3.54 [2.53, 4.56]	A arm: .03, p = .9, [6.7, 6.3] A waist: 1.3, p = .3, [5.3, 7.7] A ankle: 5.8, p = .02, [9.0, 4.0] M arm: .00, p = .99, [6.5, 6.5]	A arm: .00 A waist: .02 A ankle: .53 M arm: .00
Stroke (R)	4.96 [3.88, 6.03]	4.38 [3.50, 5.26]	4.83 [3.67, 5.99]	3.28 [2.83, 3.74] †	4.23 [3.34, 5.13]	A arm: 2.1, *p* = .15, [5.7, 3.2] A waist: .08, p = .8, [4.7, 4.2] A ankle: 1.3, *p* = .25, [5.5, 3.5] M arm: 4.1, p = .04, [2.7, 6.2]	A arm: .3 A waist: .01 A ankle: .18 M arm: .59
Multi-sit-to-stand							
Healthy	6.66 [5.47, 7.85]	5.14 [3.66, 6.62]	1.02 [0.97, 1.07]*	3.14 [2.49, 3.79] †	5.36 [3.73, 6.98]	A arm: 1.5, p = .2, [12.1, 8.9] A waist: .1, p = .8, [10.1, 10.9] A ankle: 15.7, *p* < .001, [5.5,15.5] M arm: 4.16, p = .04, [7.8, 13.2]	A arm: .08 A waist: .01 A ankle: .83 M arm: .22
iSCI	3.88 [2.53, 5.23]	1.23 [0.90, 1.56]*	1.00 [.001, .001]*	1.92 [1.34, 2.49] †	2.68 [2.26, 3.10]	A arm: 2.8, p = .1, [10.5, 6.5] A waist: 10.7, p = .001, [4.6, 12.4] A ankle: 12.9, p < .001, [4.5, 12.5] M arm: 4.9, *p* = .03, [5.8,11.1]	A arm: .19 A waist: .71 A ankle: .86 M arm: .33
Stroke (L)	4.30 [3.02, 5.58]	3.50 [1.49, 5.51]	1.00 [.001, .001]*	1.38 [1.26, 1.51] †	3.16 [2.23, 4.10]	A arm: 3.7, p = .06, [8.5, 4.5] A waist: .03, p = .9, [6.7, 6.3] A ankle: 9.5, p = .002, [3.5, 9.5] M arm: 8.3, p = .004, [3.5, 9.5]	A arm: .34 A waist: .00 A ankle: .86 M arm: .75
Stroke (R)	2.06 [0.45, 3.68]	3.85 [2.17, 5.53]	1.00 [.001, .001]*	1.50 [1.17, 1.83] †	3.29 [2.80, 3.78]	A arm: 3, p = .08, [3.0,6.0] A waist: 1.3, p = .25, [5.5, 3.5] A ankle: 6.0, *p* = .01, [2.5, 6.5] M arm: 5.3, p = .02, [2.5, 6.5]	A arm: .43 A waist: .18 A ankle: .86 M arm: .76

†*p* < 0.05 (Metria-IH1); *p < 0.016 (ActiGraph)

### Demographics

In total, 28 participants completed the study. All participants completed the protocol successfully within 60 min. There were no adverse events. The descriptive statistics of the study population is provided in Table 1.

### Activity type classification (Borg RPE)

Activities were classified as sedentary (lying, sitting and standing), low intensity (50 step walking) and high intensity (6MWT and multi sit-to-stand) (Table 1, Fig. 1(e)) based on the participants self-reported perceived exertion. Consistent with literature, individuals with stroke and iSCI rated their activities at a higher exertion level than healthy controls [41].

### Sedentary activities

**Fig. 2:**
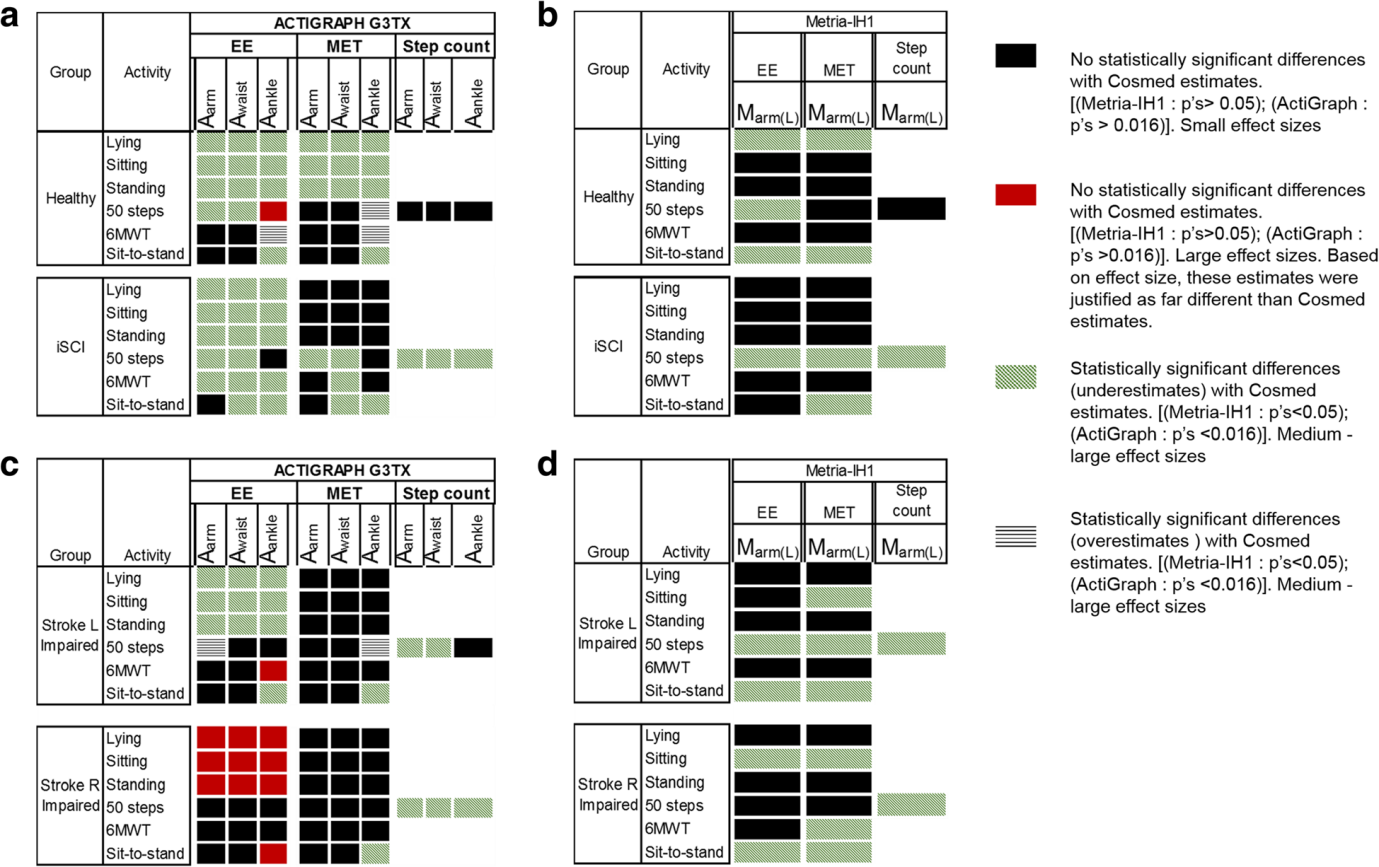
A visual comparison of the validity maps for bias in the estimated EE and MET. Estimates from both the devices in comparison to the Cosmed for the spectrum of activities performed in Healthy, iSCI and stroke groups. (**a**,**c**) Validity map for estimates from ActiGraph wG3TX-BT’s located at waist, ankle and upper arm on the right side (using ActiLife’s SPA), (**b**,**d**) Validity map for estimates from Matria-IH1 located at back side of the left upper arm (using Metria-IH1’s Senseware platform SPA). The effect of sensor location on the outcome estimates (EE, MET and step count) when using SPAs for the population with stroke and iSCI from our sample is visually summarized in the map

EE estimated by the ActiLife were significantly lower (*p* < .016; E^2^ > 0.7) than the Cosmed for all sedentary activities irrespective of the sensor locations and population studied. (Tables 2 & 3, Fig. 2a).

No significant differences were observed for EE estimates between Metria-IH1 and Cosmed for all sedentary activities in iSCI group (*p* > .05; E^2^ ≤ .3) and stroke groups with left impairment (*n* = 6) (p > .05; E^2^ < .3). In the healthy group, except for the EE during lying activity (*p* < .05; E^2^ > .7), no significant differences were observed between EE estimates from Metria-IH1 and Cosmed for other sedentary activities in the healthy controls (p > .05; E^2^ < .1); (Tables 2 & 3, Fig. 2b). The MET’s followed the same trend as EE’s.

*These results show that the sensor type and characteristics of population studied can influence the accuracy of estimates during sedentary activity when using SPA*.

### Low intensity activity

**Fig. 3:**
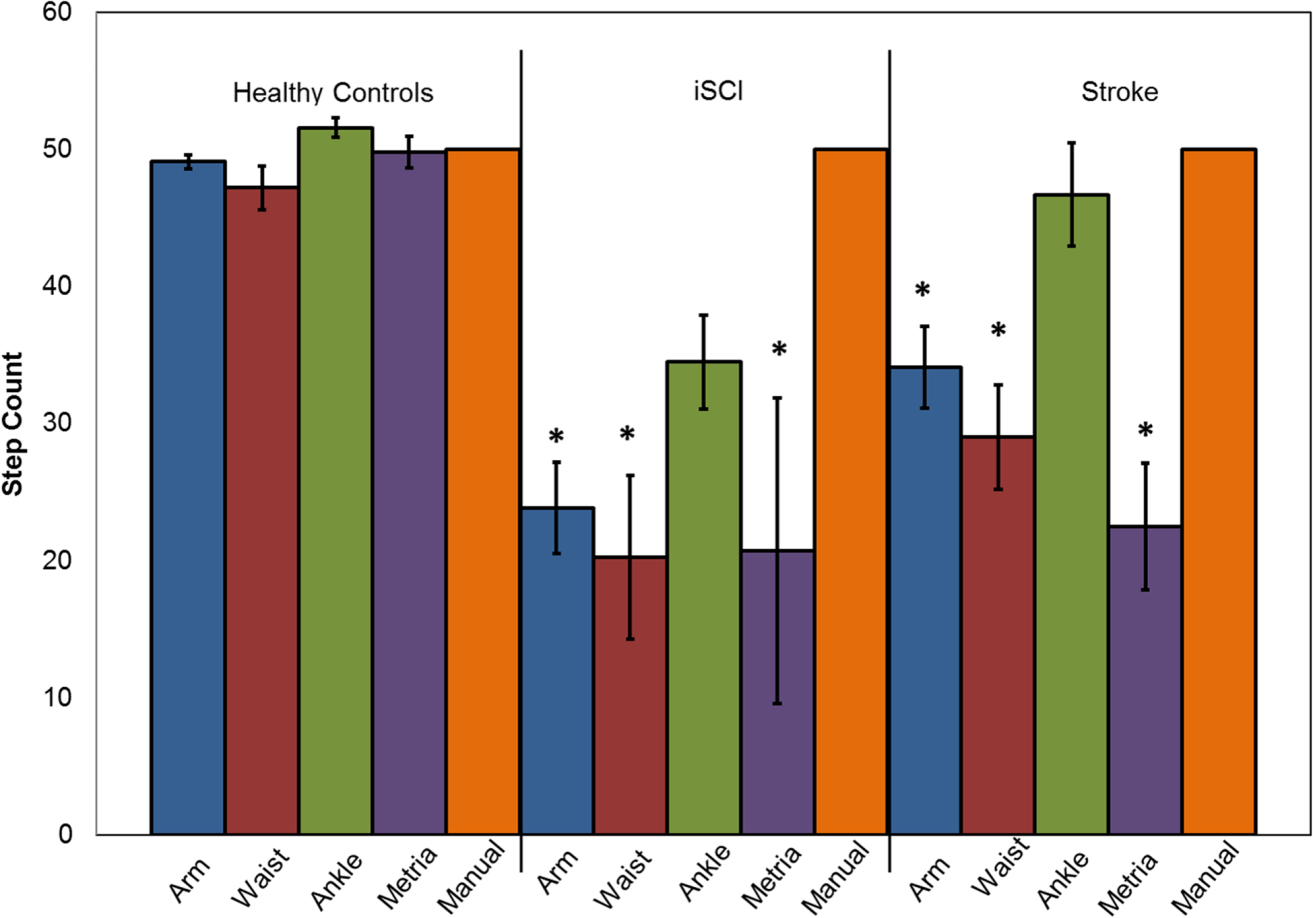
Step count estimates. Estimated step counts during the 50 step walk test from Actigraphs at arm, waist, ankle and Metria-IH1 compared to manual count (phone based manual tally) of 50 steps in healthy, SCI and Stroke. * indicates significant differences in estimated step count (*: *p* < 0.05 (Metria-IH1); *p* < 0.016(ActiGraph))

No significant differences were observed between the manually counted steps (i.e. 50 steps) and the step counts estimated by the ActiLife (irrespective of sensor locations) and Senseware (Metria-IH1) for the healthy control group (*p* > 0.016) (Fig. 3). In the group with stroke, except the ActiLife estimates for ActiGraph located at the ankle (*p* > .016) (Fig. 3), ActiGrpah’s at all other locations and the Metria-IH1 significantly underestimated the step count (*p* < 0.016). For the group with iSCI, the ActiLife (all sensor locations) and Metria-IH1, significantly under-estimated the step counts (*p* < .016) (Fig. 3). These observations with step counts are consistent with previous literature, thus benchmarking the quality of data collected in this investigation [13, 42]. These results suggest that, (i) the step count estimates from standard algorithms can be influenced by effects such as sensor location and the optimal location to place sensor for step count tracking can vary depending on the specific type of population being studied.

The EE for the 50 step walk from the ActiLife for the healthy controls and iSCI groups were significantly different than the Cosmed when placed at arm and waist locations (p < 0.016) (Table 2, Fig. 2(a), 2(b)). No statistical significant differences were observed in the ActiLife EE estimates at all placement locations for the stroke group with right side impairment (*p* > .016) (Table 2, Fig. 2(c), 2(d)).

In general, the Metria-IH1 underestimated the EE for the low intensity activity for all groups (*p* < .05; medium effect sizes) [(χ^2^ = 7; E^2^ = .37)_Healthy_; (χ^2^ = 5.8; E^2^ = .39)_iSCI_; (χ^2^ = 6.5; E^2^ = .41)_Stroke (L)_] (Tables 2 & 3, Fig. 2(b)).

Overall the MET showed similar validity trends as EE for iSCI and stroke groups (Fig. 2). For the healthy group, except the MET from ActiLife at ankle, MET’s from all other locations showed same trend as EE.

*These results highlight two main observations for low intensity activity; (1) sensor location that may be valid for estimating step count may not be valid for estimating metrics like EE/MET, and (2) using SPAs for estimating physical activity metrics in population with neurologic impairment like stroke and iSCI may yield inaccurate estimates*.

### High intensity activity

#### Six-minute walk test

In comparison to Cosmed estimates, no statistical significant differences were observed in the ActiLife EE estimates for the healthy controls at arm and waist locations (*p* > 0.016) (Table 2; Fig. 2(a)). However the ActiLife EE estimates from ActiGraph at ankle was significantly over estimated in comparison to cosmed (*p* < .016; E^2^ = .58) (Table 2). These results for the healthy group are in contrast to the EE trends from 50 step walk test. Thus, the activity intensity and duration can influence the accuracy of estimates from SPA even in a healthy group.

Similarly, for the 6MWT in the stroke group with right side impairment, in comparison to cosmed estimates, no significant difference was observed for the ActiLife EE estimates from ActiGraph’s at arm, waist and ankle (*p* > .016) (Table 2; Fig. 2(c)). However, for the stroke group with left side impairment, the EE estimates from ActiGraph at the right ankle, was only near significant with moderate effect size (*p* = 0.04; E^2^ = 0.39). Further, diminished effect sizes were observed for the ActiGraphs from the right side of the body for the group with left impaired. Similarly, it was observed that for the stroke group with right side impairment, the EE estimates from Metria-IH1 located at the left upper arm, was significantly different in comparison to Cosmed (*p* = 0.08; E^2^ = 0.43) (Table 2; Fig. 2(d)). This raises the possibility that when using fusion based sensor type to study EE (Metria-IH1- SenseWear), placing the sensors on the side of impairment may be a conservative approach for estimating EE. Indeed similar observations regarding side of impairment and EE estimate has been reported in stroke literature [43].

ActiLife EE estimates from all sensor locations for the group with iSCI, were significantly different (*p* < .016) in comparison to cosmed. The effect sizes ranging between medium to large (Tables 2 & 3, Fig. 2(a)). This shows that, the SPAs might not yield accurate results for EE in iSCI population. Based on effect size for EE estimates from 6MWT, the waist seems to be a non-optimal location for placing ActiGraph sensor for iSCI group. A potential reason for this could be the reduced walking speed in the iSCI group (0.5(0.22) m/s).

In all the groups studied, the Metria-IH1 EE estimates showed no statistical significant difference with Cosmed estimates (*p* > .05) [(χ^2^ = 1.0; E^2^ = .05)_Healthy_; (χ^2^ = .04; E^2^ = .00)_iSCI_; (χ^2^ = .03; E^2^ = .00)_Stroke(L)_; (χ^2^ = 3.0; E^2^ = .43)_Stroke (R)_].

A rationale for Metria-IH1 to have performed well with iSCI sample studied despite using SPA based off of healthy controls could be that the SenseWear fuses a galvanic skin response (GSR) sensor information among others to estimate EE and MET. We speculate that the excess exertion during the prolonged 6MWT could have increased skin conductance due to increased sweating causing the Metria-IH1’s fusion SPA to over-estimate EE values and thus producing values closer to Cosmed. It is known that individuals with cervical iSCI are compromised in their autonomic nervous system functioning which could lead to reflex sweating [44]. A majority (75%) of the iSCI group in this study had injury at cervical level where unilateral hyperhidrosis and reflex sweating is a reported phenomenon [45]. Indeed, literature suggests that higher exertion could lead to higher skin conductance [46]. The group with iSCI reported higher physical exertion during the low and high intensity activities (Table 1).

#### Multi sit-to-stand

In comparison to Cosmed EE estimates, no significant differences were observed for the ActiLife EE estimates from ActiGraphs (Table 2; Fig. 2), (i) at arm and waist for healthy group, (ii) waist for the stroke groups and (iii) arm for the iSCI group. Irrespective of side of stroke impairment, the sensors at waist seems to be a desirable location to estimate EE during sit-to-stand task in stroke group. Except for the EE estimates for iSCI group, the Metria-IH underestimated the EE during the multi sit-to-stand task in stroke and healthy groups. The MET metrics followed a similar trend as the EE for healthy and stroke groups during 6MWT and multi sit-to-stand.

*These results could suggest that, (i) the choice of sensor location could be dependent on activity type and outcome metric of interest and (ii) impairment conditions can significantly impact outcome metric estimated by SPA*.

## Discussion

This study systematically analyzed the influence of four factors, namely, (i) choice of sensor type ((ActiGraph wG3TX-BT using ActiLife SPA) and Metria-IH1 -Senseware fusion based SPA), (ii) sensor location (ActiGraph wG3TX-BT at arm, waist and ankle and Metria-IH1 at arm) (iii) the activity characteristics and (iv) population effects (healthy, iSCI(ambulatory), Stroke) on the validity of three physical activity outcome metrics estimated by SPAs. Overall it was found that the physical activity metrics (EE, MET and step count) estimated by SPAs could be influenced significantly by these factors across the spectrum of activity levels studied.

Consistent with previous literature [13], the SPAs from both the sensors estimated the step count metric accurately in the control group, irrespective of sensor location (ActiLife) and type. However, observations from our data showed that, the estimates by these SPAs for EE and MET significantly diverged from the gold standard estimates at all activity levels. The sensor location, sensor type and activity type seemed to influence the EE and MET estimates provided by SPA even in healthy controls group. For instance, sensor placed at arm and waist seems to estimate EE and MET better during low and high intensity activities in comparison to sensor located at the ankle (Fig. 2(a)). Similar observations on healthy individuals have been reported in literature [47]. Benchmarking with previous literature serves as a support for the consistency of data collected and analyzed in this study.

The SPAs estimation for the activity data collected from the study groups with iSCI and stroke were mediocre. For instance, in the group with iSCI, irrespective of sensor location and sensor type, the step counts estimates were inaccurate. Further, based on the ActiLife SPA estimates for iSCI, irrespective of sensor location and activity type studied, most of the EE estimates were significant deviants from the estimates produced by the designated gold standards. However, overall, based on a subjective comparison, the Sensewear performed relatively better for estimation in the iSCI and stroke groups [48]. On the same lines, in comparison to ActiLife, the fusion algorithm from Senseware seemed to perform relatively better for EE and MET estimation during the studied sedentary tasks.

Finally, the trends from the multi sit-to-stand activity showed that, the sensor location should be chosen based on the nature of the activity type being studied. For instance, the arm and/or waist seems to be a desirable spot to estimate EE during sit-to-stand task for the healthy controls and the stroke group. For the iSCI group, sensors located at the arm seemed to capture the EE estimates well.

Overall, there are three possible reasons for such divergences observed in validity while using SPAs based off of healthy population to estimate outcomes from wearable sensors in stroke and iSCI.

Firstly, it is possible that the SPAs estimated outcome metrics based on the movement signature and acceleration thresholds empirically derived off of database collected from healthy population. These standard acceleration threshold values from healthy are far higher than those observed in neurological population [1, 2, 4, 49]. Individuals with iSCI and stroke in general walk with a gait speed much slower in comparison to healthy population [50, 51] and also use assistive devices. The average gait speeds during 6MWT for the different participant groups from our study sample were, 1.7(0.23) m/s, 0.5(0.22) m/s and 0.9(0.20) m/s for the healthy, iSCI and stroke respectively. Decreased speed and gait signatures while performing physical activities changes the acceleration thresholds leading to underestimates in step counts (an additional file shows this in more detail [see Additional file 2: Figure S1]). Secondly, sensors placed at different body locations (arm, waist and ankle) unfold different acceleration signatures (cut points) for a given activity type, due the dynamics constraint in motion between different body segments and sensor location [13, 52]. Finally, it is reasonable to expect that neurologic impairment like stroke and iSCI alters the metabolic profile. Hence empirically derived EE models based off of healthy controls may not work for this population [11, 53–55]. Thus the effect of choice of sensor type, sensor location and activity characteristics seems to be additional factor that needs consideration on top of population specific differences which influence the deviation of the estimates derived using SPAs.

We observed from our results that, for the stroke group, both the Actilife and Metria-IH1 (Senseware) SPAs performed relatively well when placed on the impaired side as opposed to unimpaired side for EE and MET estimation (Fig. 2(c), 2(d)). Overall, we also observed that despite using the standard SPAs some of the EE and MET estimates turned out to produce valid estimates for the group with stroke and iSCI.

As far as this sample data goes, there are a few possible explanations for above observations. One possible speculation from a pathophysiological angle could be the asymmetric sweat response that has been reported in individuals with stroke due to compromise in functioning of their autonomic nervous system [56–58].It is possible that the increased sweating on the paretic side could have improved the skin conductance, leading to the GSR sensor in Metria-IH1's (SenseWear) SPA overestimating the EE values, thus leading to values close to the Cosmed. Literature suggests that a higher physical exertion level can lead to higher skin conductance [46]. Indeed self-reported physical exertion levels were higher for both the stroke and iSCI groups compared to healthy controls (Table 1). We speculate that a similar phenomenon could have led to overall better EE and MET estimates for the iSCI group while using Sensewear [44]. However, it is promising to find support for this observation in literature [43]. We did not record data regarding the sweat rate or skin temperature in this study. Nor did we have access to the Sensewear’s SPA to tease out the weightage given to GSR data in their fusion algorithm. These aspects require more work and specifically designed study to understand the influence of such factors are warranted.

We also suspect that since the participants from the stroke group in our study had mild-moderate gait impairments (mean gait speed was relatively higher 0.9 (0.20) m/s during 6MWT), the acceleration threshold was sufficient to create enough count points for the SPAs to produce better estimates. Similar observations have been noted in literature [42]. We can only speculate that this could be the reason that the ActiLife SPAs estimates showed validity for some of the activities while used for the group with stroke.

Additionally from a sensor capability stand point, a potential reason for this could be that unlike the ActiLife (ActiGraph sampled at 30 Hz) the Metria-IH1’s SPA estimates EE and MET by fusing information from multiple on board sensor modules (overall sampling of all sensors at 5000 data points per minute in which accelerometer alone sampled at 32 Hz), such as, tri-axial accelerometer, near body skin temperature sensor and galvanic skin response in addition to customized participant specific information such as smoking behavior, and anthropometrics. Indeed literature shows that sensor fusion based approaches yield better measurements of metrics, contingent upon the quality of the sensors [2, 48, 59–61].

In summary, there were two main findings and recommendations form this pilot investigation, (i) the sensor type, sensor location, activity characteristics and the population studied influences the accuracy of estimation of physical activity metrics derived using SPAs: implementing advanced techniques like machine learning and data fusion to create customized population specific algorithms to estimate physical activity metrics in individuals with neurologic impairments such as iSCI and stroke has the potential to improve the reliability & accuracy and (ii) comprehensive validations including all outcome metrics (EE, MET and step counts) at different activity intensity level is recommended for validation of wearable sensors used in rehabilitation. These findings are also in consensus with findings from literature studying validity of wearable sensor estimates in different groups [13, 14, 52, 62–65].

## Limitations

Despite producing some novel and clinically useful information, this investigation has many limitations. The small sample size limits the extent of generalizability of our findings. However, the sample size in our study is comparable to other studies in this literature [66] and our findings were supported with observations from previous literature. The sedentary activities were recorded only for bouts of 2 min each. It is not clear if the same trends would hold when data is gathered for a different time scale. However, to justify our choice of this time scale, it is reasonable to assume that there is value in studying such small bouts (< 2 min) of sedentary activities as such low/high intensity activity occur through-out the day in community setting. Future studies with a larger sample size and includes other types of neurological impairments is recommended to explore the individual influence of each of the factors for population specific conditions on the outcome variables in laboratory as well as free living conditions.

### Conclusions

On one hand the inferences and information from our results highlight the need to practice cautious decision making while choosing wearable sensor types and mounting locations for activity measurement in neurologic rehabilitation. Whilst on the other hand, implementing customized algorithms using advanced methods like machine learning and data fusion methodologies for estimating outcomes using wearables in individuals neurological impairments like stroke and iSCI could be beneficial [2]. We maintain that, incorporating the combined effect of choice of sensor type used, location of placement, and the activity intensity being studied, to algorithms estimating outcome metrics from wearable devices may yield reliable physical activity metrics both in in-patient and out-patient environments.

## Additional files

Additional file 1: tables with post-hoc analysis. Description:**Table ST1.** Games-Howell multiple comparison post-hoc test to assess absolute agreement (EE estimates in healthy control group). **Table ST2.** Games-Howell multiple comparison post-hoc test to assess absolute agreement (MET estimates in healthy control group). **Table ST3.** Games-Howell multiple comparison post-hoc test to assess absolute agreement (EE estimates in iSCI control group). **Table ST4.** Games-Howell multiple comparison post-hoc test to assess absolute agreement (MET estimates in iSCI control group). **Table ST5.** Games-Howell multiple comparison post-hoc test to assess absolute agreement (EE estimates in stroke group with right impairment). **Table ST6.** Games-Howell multiple comparison post-hoc test to assess absolute agreement (MET estimates in stroke group with right impairment). **Table ST7.** Games-Howell multiple comparison post-hoc test to assess absolute agreement (EE estimates in stroke group with left impairment). **Table ST8.** Games-Howell multiple comparison post-hoc test to assess absolute agreement (MET estimates in stroke group with left impairment). (DOCX 41kb)

Additional file 2: **FigureS1.** Sample acceleration data. Acceleration (triaxial) from ActiGraphs strapped to arm, waist and ankle during a 50 step walk test from a representative healthy, iSCI and stroke participants. Note: The duration for the 50 step walk test is different across the groups. (PNG 93 kb)

## Abbreviations

6MWT: Six-minute walk test
EE: Energy expenditure
iSCI: Incomplete spinal cord injury
MET: Metabolic equivalent
SPA: Standard proprietary algorithm
Stroke(L): Stroke with left impairment
Stroke(R): Stroke with right impairment
