# Supplementary figures and images for: Variables influencing wearable sensor outcome estimates in individuals with stroke and incomplete spinal cord injury: a pilot investigation validating two research grade sensors
Source: J Neuroeng Rehabil. 2018 Mar 13;15:19. doi: 10.1186/s12984-018-0358-y (PMC5850975; doi:10.1186/s12984-018-0358-y)

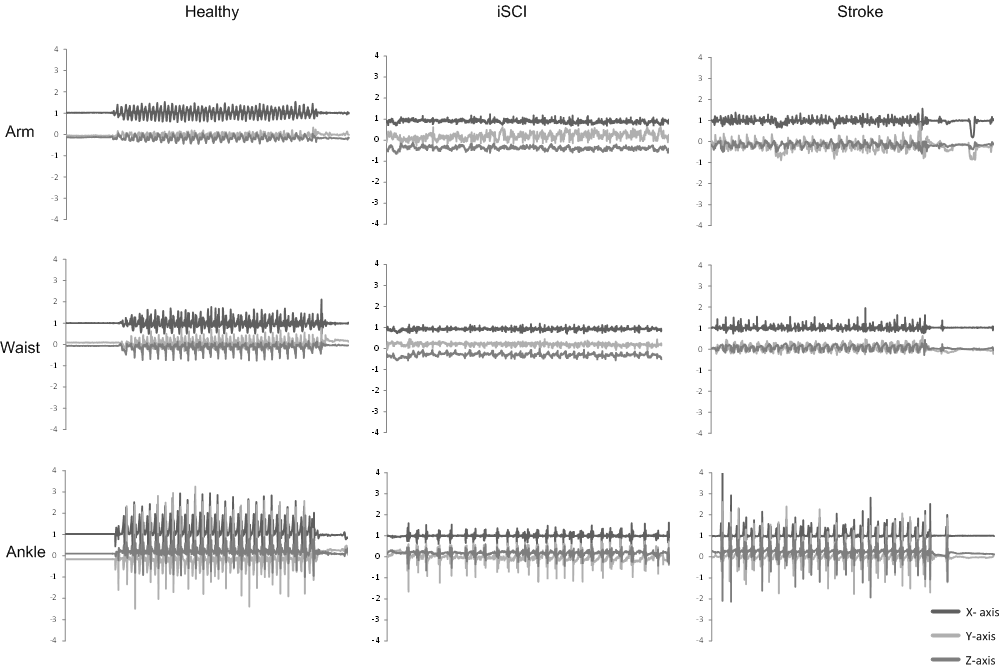

Supplement: Supplementary file 1 — tables with post-hoc analysis. Description:Table ST1. Games-Howell multiple comparison post-hoc test to assess absolute agreement (EE estimates in healthy control group). Table ST2. Games-Howell multiple comparison post-hoc test to assess absolute agreement (MET estimates in healthy control group). Table ST3. Games-Howell multiple comparison post-hoc test to assess absolute agreement (EE estimates in iSCI control group). Table ST4. Games-Howell multiple comparison post-hoc test to assess absolute agreement (MET estimates in iSCI control group). Table ST5. Games-Howell multiple comparison post-hoc test to assess absolute agreement (EE estimates in stroke group with right impairment). Table ST6. Games-Howell multiple comparison post-hoc test to assess absolute agreement (MET estimates in stroke group with right impairment). Table ST7. Games-Howell multiple comparison post-hoc test to assess absolute agreement (EE estimates in stroke group with left impairment). Table ST8. Games-Howell multiple comparison post-hoc test to assess absolute agreement (MET estimates in stroke group with left impairment). (DOCX 41kb) [file 12984_2018_358_MOESM1_ESM.png]
