# Supplementary material for: Variables influencing wearable sensor outcome estimates in individuals with stroke and incomplete spinal cord injury: a pilot investigation validating two research grade sensors
Source: J Neuroeng Rehabil. 2018 Mar 13;15:19. doi: 10.1186/s12984-018-0358-y (PMC5850975; doi:10.1186/s12984-018-0358-y)
Supplement: Supplementary file 2 — FigureS1. Sample acceleration data. Acceleration (triaxial) from ActiGraphs strapped to arm, waist and ankle during a 50 step walk test from a representative healthy, iSCI and stroke participants. Note: The duration for the 50 step walk test is different across the groups. (PNG 93 kb) [file 12984_2018_358_MOESM2_ESM.docx]

**Supplementary tables (Post-hoc analysis)**

Table ST1. Games-Howell multiple comparison post-hoc test to assess absolute agreement (EE estimates in healthy control group).

| Spectrum of activities | Gold standard EE estimate | SPA derived EE estimates from different locations | Mean Difference (I-J) | Std. Error | Sig. | 98.4% Confidence Interval | |
| --- | --- | --- | --- | --- | --- | --- | --- |
|  |  |  |  |  |  | Lower Bound | Upper Bound |
| Lying | Cosmed K4B2 | A _arm (right)_ | 3.13 | 0.24 | .000* | 2.18 | 4.10 |
|  |  | A _waist (right)_ | 3.17 | 0.23 | .000* | 2.22 | 4.14 |
|  |  | A _ankle (right)_ | 3.14 | 0.23 | .000* | 2.19 | 4.10 |
|  |  | Metria _(M arm (left))_ | 1.16 | 0.25 | .004 | 0.20 | 2.13 |
| Sitting | Cosmed K4B2 | A _arm (right)_ | 2.05 | 0.12 | .000* | 1.56 | 2.55 |
|  |  | A _waist (right)_ | 2.08 | 0.12 | .000* | 1.59 | 2.57 |
|  |  | A _ankle (right)_ | 2.07 | 0.12 | .000* | 1.59 | 2.57 |
|  |  | Metria _(M arm (left))_ | -0.25 | 0.21 | .768 | -1.02 | 0.53 |
| Standing | Cosmed K4B2 | A _arm (right)_ | 1.92 | 0.13 | .000* | 1.40 | 2.46 |
|  |  | A _waist (right)_ | 1.94 | 0.13 | .000* | 1.41 | 2.47 |
|  |  | A _ankle (right)_ | 1.93 | 0.13 | .000* | 1.41 | 2.47 |
|  |  | Metria _(M arm (left))_ | -0.16 | 0.22 | .944 | -0.97 | 0.64 |
| 50 steo walk | Cosmed K4B2 | A _arm (right)_ | 0.96 | 0.32 | .052 | -0.18 | 2.11 |
|  |  | A _waist (right)_ | 1.20 | 0.32 | .011* | 0.05 | 2.35 |
|  |  | A _ankle (right)_ | -1.22 | 0.44 | .082 | -2.82 | 0.37 |
|  |  | Metria _(M arm (left))_ | 0.82 | 0.27 | .059 | -0.20 | 1.83 |
| 6MWT | Cosmed K4B2 | A _arm (right)_ | 0.73 | 0.98 | .941 | -3.05 | 4.52 |
|  |  | A _waist (right)_ | 0.41 | 0.95 | .992 | -3.25 | 4.08 |
|  |  | A _ankle (right)_ | -3.09 | 0.71 | .004* | -5.72 | -0.47 |
|  |  | Metria _(M arm (left))_ | 0.65 | 0.51 | .706 | -1.17 | 2.47 |
| Multi sit-to-stand | Cosmed K4B2 | A _arm (right)_ | -1.13 | 1.19 | .875 | -5.40 | 3.15 |
|  |  | A _waist (right)_ | 0.77 | 1.22 | .968 | -3.62 | 5.16 |
|  |  | A _ankle (right)_ | 6.64 | 0.80 | .000* | 3.36 | 9.92 |
|  |  | Metria _(M arm (left))_ | 3.24 | 0.84 | .017* | -0.04 | 6.53 |
| *. The mean difference is significant at the 0.016 level. EE: Energy expenditure; SPA: Standard proprietary algorithm; A _arm (right)_: ActiGraph G3Tx mounted at right arm; A _waist (right)_: ActiGraph G3Tx mounted at right waist; A _ankle (right)_: ActiGraph G3Tx mounted at right ankle; Metria _(M arm (left))_: Metria-IH1 mounted upper left arm; 6MWT: six minute walk test. | | | | | | | |

Table ST2. Games-Howell multiple comparison post-hoc test to assess absolute agreement (MET estimates in healthy control group).

| Spectrum of activities | Gold standard MET estimate | SPA derived MET estimates from different locations | Mean Difference (I-J) | Std. Error | Sig. | 98.4% Confidence Interval | |
| --- | --- | --- | --- | --- | --- | --- | --- |
|  |  |  |  |  |  | Lower Bound | Upper Bound |
| Lying | Cosmed K4B2 | A _arm (right)_ | 1.29 | 0.21 | .001* | 0.46 | 2.14 |
|  |  | A _waist (right)_ | 1.32 | 0.20 | .001* | 0.48 | 2.17 |
|  |  | A _ankle (right)_ | 1.30 | 0.20 | .001* | 0.46 | 2.14 |
|  |  | Metria _(M arm (left))_ | 0.54 | 0.23 | .201 | -0.33 | 1.41 |
| Sitting | Cosmed K4B2 | A _arm (right)_ | 0.59 | 0.14 | .014* | 0.01 | 1.18 |
|  |  | A _waist (right)_ | 0.59 | 0.14 | .014* | 0.02 | 1.18 |
|  |  | A _ankle (right)_ | 0.59 | 0.14 | .014* | 0.02 | 1.18 |
|  |  | Metria _(M arm (left))_ | -0.43 | 0.21 | .300 | -1.19 | 0.33 |
| Standing | Cosmed K4B2 | A _arm (right)_ | 0.51 | 0.13 | .018 | -0.01 | 1.03 |
|  |  | A _waist (right)_ | 0.51 | 0.13 | .018 | -0.01 | 1.03 |
|  |  | A _ankle (right)_ | 0.51 | 0.13 | .018 | -0.01 | 1.03 |
|  |  | Metria _(M arm (left))_ | -0.30 | 0.17 | .441 | -0.92 | 0.32 |
| 50 steo walk | Cosmed K4B2 | A _arm (right)_ | 0.23 | 0.35 | .959 | -1.03 | 1.50 |
|  |  | A _waist (right)_ | 0.37 | 0.35 | .823 | -0.90 | 1.65 |
|  |  | A _ankle (right)_ | -1.49 | 0.30 | .003* | -2.67 | -0.31 |
|  |  | Metria _(M arm (left))_ | 0.55 | 0.29 | .385 | -0.63 | 1.72 |
| 6MWT | Cosmed K4B2 | A _arm (right)_ | -0.01 | 0.80 | 1.000 | -2.89 | 2.87 |
|  |  | A _waist (right)_ | -0.16 | 0.67 | .999 | -2.64 | 2.33 |
|  |  | A _ankle (right)_ | -2.65 | 0.68 | .01* | -5.14 | -0.16 |
|  |  | Metria _(M arm (left))_ | 0.17 | 0.67 | .999 | -2.31 | 2.64 |
| Multi sit-to-stand | Cosmed K4B2 | A _arm (right)_ | -1.30 | 0.89 | .601 | -4.53 | 1.93 |
|  |  | A _waist (right)_ | 0.22 | 0.97 | .999 | -3.26 | 3.70 |
|  |  | A _ankle (right)_ | 4.33 | 0.72 | .001* | 1.36 | 7.32 |
|  |  | Metria _(M arm (left))_ | 2.22 | 0.77 | .086 | -0.77 | 5.20 |
| *. The mean difference is significant at the 0.016 level. MET: Metabolic equivalent; SPA: Standard proprietary algorithm; A _arm (right)_: ActiGraph G3Tx mounted at right arm; A _waist (right)_: ActiGraph G3Tx mounted at right waist; A _ankle (right)_: ActiGraph G3Tx mounted at right ankle; Metria _(M arm (left))_: Metria-IH1 mounted upper left arm; 6MWT: six minute walk test. | | | | | | | |

Table ST3. Games-Howell multiple comparison post-hoc test to assess absolute agreement (EE estimates in iSCI control group).

| Spectrum of activities | Gold standard EE estimate | SPA derived EE estimates from different locations | Mean Difference (I-J) | Std. Error | Sig. | 98.4% Confidence Interval | |
| --- | --- | --- | --- | --- | --- | --- | --- |
|  |  |  |  |  |  | Lower Bound | Upper Bound |
| Lying | Cosmed K4B2 | A _arm (right)_ | 1.58 | 0.14 | .000* | 0.97 | 2.21 |
|  |  | A _waist (right)_ | 1.58 | 0.14 | .000* | 0.97 | 2.21 |
|  |  | A _ankle (right)_ | 1.58 | 0.14 | .000* | 0.97 | 2.21 |
|  |  | Metria _(M arm (left))_ | -0.27 | 0.23 | .764 | -1.16 | 0.61 |
| Sitting | Cosmed K4B2 | A _arm (right)_ | 1.43 | 0.10 | .000* | 1.00 | 1.87 |
|  |  | A _waist (right)_ | 1.47 | 0.10 | .000* | 1.03 | 1.92 |
|  |  | A _ankle (right)_ | 1.47 | 0.10 | .000* | 1.04 | 1.92 |
|  |  | Metria _(M arm (left))_ | -0.44 | 0.21 | .302 | -1.30 | 0.41 |
| Standing | Cosmed K4B2 | A _arm (right)_ | 1.71 | 0.17 | .000* | 0.97 | 2.46 |
|  |  | A _waist (right)_ | 1.73 | 0.17 | .000* | 0.98 | 2.49 |
|  |  | A _ankle (right)_ | 1.73 | 0.17 | .000* | 0.98 | 2.49 |
|  |  | Metria _(M arm (left))_ | -0.44 | 0.40 | .800 | -2.05 | 1.17 |
| 50 step walk | Cosmed K4B2 | A _arm (right)_ | 2.25 | 0.51 | .007* | 0.25 | 4.27 |
|  |  | A _waist (right)_ | 2.88 | 0.48 | .001* | 0.91 | 4.86 |
|  |  | A _ankle (right)_ | 0.81 | 1.09 | .942 | -3.63 | 5.25 |
|  |  | Metria _(M arm (left))_ | 0.81 | 0.92 | .899 | -2.83 | 4.46 |
| 6MWT | Cosmed K4B2 | A _arm (right)_ | 2.87 | 0.97 | .067 | -0.76 | 6.49 |
|  |  | A _waist (right)_ | 4.15 | 0.75 | .002* | 1.08 | 7.23 |
|  |  | A _ankle (right)_ | 2.88 | 0.97 | .064 | -0.73 | 6.50 |
|  |  | Metria _(M arm (left))_ | -0.39 | 1.17 | .997 | -4.81 | 4.04 |
| Multi sit-to-stand | Cosmed K4B2 | A _arm (right)_ | -0.82 | 0.98 | .912 | -5.05 | 3.41 |
|  |  | A _waist (right)_ | 2.29 | 0.45 | .002* | 0.57 | 4.01 |
|  |  | A _ankle (right)_ | 3.77 | 0.27 | .000* | 2.57 | 4.97 |
|  |  | Metria _(M arm (left))_ | 0.49 | 0.88 | .979 | -3.24 | 4.21 |
| *. The mean difference is significant at the 0.016 level. EE: Energy expenditure; SPA: Standard proprietary algorithm; A _arm (right)_: ActiGraph G3Tx mounted at right arm; A _waist (right)_: ActiGraph G3Tx mounted at right waist; A _ankle (right)_: ActiGraph G3Tx mounted at right ankle; Metria _(M arm (left))_: Metria-IH1 mounted upper left arm; 6MWT: six minute walk test; iSCI: incomplete spinal cord injury. | | | | | | | |

Table ST4. Games-Howell multiple comparison post-hoc test to assess absolute agreement (MET estimates in iSCI control group).

| Spectrum of activities | Gold standard MET estimate | SPA derived MET estimates from different locations | Mean Difference (I-J) | Std. Error | Sig. | 98.4% Confidence Interval | |
| --- | --- | --- | --- | --- | --- | --- | --- |
|  |  |  |  |  |  | Lower Bound | Upper Bound |
| Lying | Cosmed K4B2 | A _arm (right)_ | 0.12 | 0.08 | .547 | -0.22 | 0.46 |
|  |  | A _waist (right)_ | 0.12 | 0.08 | .547 | -0.22 | 0.46 |
|  |  | A _ankle (right)_ | 0.12 | 0.08 | .547 | -0.22 | 0.46 |
|  |  | Metria _(M arm (left))_ | -0.06 | 0.09 | .956 | -0.42 | 0.29 |
| Sitting | Cosmed K4B2 | A _arm (right)_ | 0.04 | 0.07 | .960 | -0.26 | 0.35 |
|  |  | A _waist (right)_ | 0.04 | 0.07 | .960 | -0.26 | 0.35 |
|  |  | A _ankle (right)_ | 0.04 | 0.07 | .960 | -0.26 | 0.35 |
|  |  | Metria _(M arm (left))_ | -0.18 | 0.09 | .281 | -0.50 | 0.14 |
| Standing | Cosmed K4B2 | A _arm (right)_ | 0.22 | 0.07 | .108 | -0.11 | 0.55 |
|  |  | A _waist (right)_ | 0.22 | 0.07 | .108 | -0.11 | 0.55 |
|  |  | A _ankle (right)_ | 0.22 | 0.07 | .108 | -0.11 | 0.55 |
|  |  | Metria _(M arm (left))_ | -0.16 | 0.17 | .891 | -0.86 | 0.55 |
| 50 step walk | Cosmed K4B2 | A _arm (right)_ | 1.22 | 0.21 | .001* | 0.43 | 2.02 |
|  |  | A _waist (right)_ | 1.40 | 0.17 | .001* | 0.62 | 2.19 |
|  |  | A _ankle (right)_ | 0.34 | 0.53 | .965 | -1.90 | 2.58 |
|  |  | Metria _(M arm (left))_ | 0.72 | 0.44 | .506 | -1.07 | 2.51 |
| 6MWT | Cosmed K4B2 | A _arm (right)_ | 1.48 | 0.52 | .084 | -0.48 | 3.43 |
|  |  | A _waist (right)_ | 2.18 | 0.35 | .002* | 0.68 | 3.69 |
|  |  | A _ankle (right)_ | 0.57 | 0.68 | .917 | -2.12 | 3.25 |
|  |  | Metria _(M arm (left))_ | -0.01 | 0.66 | 1.000 | -2.58 | 2.55 |
| Multi sit-to-stand | Cosmed K4B2 | A _arm (right)_ | -1.20 | 0.60 | .341 | -3.75 | 1.35 |
|  |  | A _waist (right)_ | 1.44 | 0.23 | .000* | 0.59 | 2.30 |
|  |  | A _ankle (right)_ | 1.68 | 0.18 | .000* | 0.87 | 2.49 |
|  |  | Metria _(M arm (left))_ | 0.76 | 0.30 | .142 | -0.38 | 1.91 |

*. The mean difference is significant at the 0.016 level. MET: Metabolic equivalent; SPA: Standard proprietary algorithm; A _arm (right)_: ActiGraph G3Tx mounted at right arm; A _waist (right)_: ActiGraph G3Tx mounted at right waist; A _ankle (right)_: ActiGraph G3Tx mounted at right ankle; Metria _(M arm (left))_: Metria-IH1 mounted upper left arm; 6MWT: six minute walk test; iSCI: incomplete spinal cord injury.

Table ST5. Games-Howell multiple comparison post-hoc test to assess absolute agreement (EE estimates in stroke group with right impairment).

| Spectrum of activities | Gold standard EE estimate | SPA derived EE estimates from different locations | Mean Difference (I-J) | Std. Error | Sig. | 98.4% Confidence Interval | |
| --- | --- | --- | --- | --- | --- | --- | --- |
|  |  |  |  |  |  | Lower Bound | Upper Bound |
| Lying | Cosmed K4B2 | A _arm (right)_ | 1.65 | 0.21 | .016* | 0.01 | 3.29 |
|  |  | A _waist (right)_ | 1.65 | 0.21 | .016* | 0.01 | 3.29 |
|  |  | A _ankle (right)_ | 1.65 | 0.21 | .016* | 0.01 | 3.29 |
|  |  | Metria _(M arm (left))_ | 0.06 | 0.25 | .999 | -1.23 | 1.34 |
| Sitting | Cosmed K4B2 | A _arm (right)_ | 1.19 | 0.07 | .002* | 0.59 | 1.79 |
|  |  | A _waist (right)_ | 1.19 | 0.07 | .002* | 0.59 | 1.79 |
|  |  | A _ankle (right)_ | 1.19 | 0.07 | .002* | 0.59 | 1.79 |
|  |  | Metria _(M arm (left))_ | -0.38 | 0.15 | .227 | -1.21 | 0.45 |
| Standing | Cosmed K4B2 | A _arm (right)_ | 1.42 | 0.19 | .020 | -0.12 | 2.97 |
|  |  | A _waist (right)_ | 1.42 | 0.19 | .020 | -0.12 | 2.97 |
|  |  | A _ankle (right)_ | 1.42 | 0.19 | .020 | -0.12 | 2.97 |
|  |  | Metria _(M arm (left))_ | -0.17 | 0.23 | .940 | -1.38 | 1.03 |
| 50 step walk | Cosmed K4B2 | A _arm (right)_ | -0.08 | 0.75 | 1.000 | -4.50 | 4.33 |
|  |  | A _waist (right)_ | 0.74 | 0.46 | .543 | -1.51 | 2.99 |
|  |  | A _ankle (right)_ | -0.23 | 0.98 | .999 | -6.62 | 6.17 |
|  |  | Metria _(M arm (left))_ | 0.84 | 0.44 | .404 | -1.30 | 2.97 |
| 6MWT | Cosmed K4B2 | A _arm (right)_ | -0.69 | 1.10 | .964 | -6.23 | 4.84 |
|  |  | A _waist (right)_ | 0.17 | 0.93 | 1.000 | -4.32 | 4.65 |
|  |  | A _ankle (right)_ | -0.49 | 1.09 | .989 | -5.95 | 4.96 |
|  |  | Metria _(M arm (left))_ | 1.71 | 0.76 | .299 | -2.45 | 5.87 |
| Multi sit-to-stand | Cosmed K4B2 | A _arm (right)_ | 1.86 | 0.89 | .348 | -2.96 | 6.68 |
|  |  | A _waist (right)_ | -0.61 | 1.00 | .966 | -6.36 | 5.14 |
|  |  | A _ankle (right)_ | 4.54 | 0.49 | .004* | 1.50 | 7.57 |
|  |  | Metria _(M arm (left))_ | 2.65 | 0.52 | .021* | -0.19 | 5.50 |
| *. The mean difference is significant at the 0.016 level. EE: Energy expenditure; Spa: Standard proprietary algorithm; A _arm (right)_: ActiGraph G3Tx mounted at right arm; A _waist (right)_: ActiGraph G3Tx mounted at right waist; A _ankle (right)_: ActiGraph G3Tx mounted at right ankle; Metria _(M arm (left))_: Metria-IH1 mounted upper left arm; 6MWT: six minute walk test. | | | | | | | |

Table ST6. Games-Howell multiple comparison post-hoc test to assess absolute agreement (MET estimates in stroke group with right impairment).

| Spectrum of activities | Gold standard MET estimate | SPA derived MET estimates from different locations | Mean Difference (I-J) | Std. Error | Sig. | 98.4% Confidence Interval | |
| --- | --- | --- | --- | --- | --- | --- | --- |
|  |  |  |  |  |  | Lower Bound | Upper Bound |
| Lying | Cosmed K4B2 | A _arm (right)_ | 0.17 | 0.13 | 0.71 | -0.85 | 1.19 |
|  |  | A _waist (right)_ | 0.17 | 0.13 | 0.71 | -0.85 | 1.19 |
|  |  | A _ankle (right)_ | 0.17 | 0.13 | 0.71 | -0.85 | 1.19 |
|  |  | Metria _(M arm (left))_ | 0.02 | 0.13 | 1.00 | -0.88 | 0.93 |
| Sitting | Cosmed K4B2 | A _arm (right)_ | -0.14 | 0.07 | 0.39 | -0.68 | 0.39 |
|  |  | A _waist (right)_ | -0.14 | 0.07 | 0.39 | -0.68 | 0.39 |
|  |  | A _ankle (right)_ | -0.14 | 0.07 | 0.39 | -0.68 | 0.39 |
|  |  | Metria _(M arm (left))_ | -0.28 | 0.07 | 0.08 | -0.72 | 0.17 |
| Standing | Cosmed K4B2 | A _arm (right)_ | 0.01 | 0.10 | 1.00 | -0.78 | 0.79 |
|  |  | A _waist (right)_ | 0.01 | 0.10 | 1.00 | -0.78 | 0.79 |
|  |  | A _ankle (right)_ | 0.01 | 0.10 | 1.00 | -0.78 | 0.79 |
|  |  | Metria _(M arm (left))_ | -0.14 | 0.10 | 0.68 | -0.82 | 0.54 |
| 50 step walk | Cosmed K4B2 | A _arm (right)_ | -0.34 | 0.43 | 0.92 | -2.60 | 1.92 |
|  |  | A _waist (right)_ | 0.18 | 0.29 | 0.97 | -1.30 | 1.66 |
|  |  | A _ankle (right)_ | -0.47 | 0.58 | 0.92 | -3.95 | 3.01 |
|  |  | Metria _(M arm (left))_ | 0.61 | 0.28 | 0.32 | -0.87 | 2.08 |
| 6MWT | Cosmed K4B2 | A _arm (right)_ | -0.73 | 0.44 | 0.52 | -2.89 | 1.43 |
|  |  | A _waist (right)_ | -0.15 | 0.40 | 0.99 | -2.07 | 1.76 |
|  |  | A _ankle (right)_ | -0.60 | 0.46 | 0.70 | -2.90 | 1.70 |
|  |  | Metria _(M arm (left))_ | 0.95 | 0.32 | 0.15 | -0.87 | 2.76 |
| Multi sit-to-stand | Cosmed K4B2 | A _arm (right)_ | 1.23 | 0.53 | 0.32 | -2.40 | 4.85 |
|  |  | A _waist (right)_ | -0.56 | 0.55 | 0.84 | -4.35 | 3.23 |
|  |  | A _ankle (right)_ | 2.29 | 0.15 | .003* | 1.06 | 3.53 |
|  |  | Metria _(M arm (left))_ | 1.79 | 0.19 | .001* | 0.83 | 2.76 |
| *. The mean difference is significant at the 0.016 level. MET: Metabolic equivalent; Spa: Standard proprietary algorithm; A _arm (right)_: ActiGraph G3Tx mounted at right arm; A _waist (right)_: ActiGraph G3Tx mounted at right waist; A _ankle (right)_: ActiGraph G3Tx mounted at right ankle; Metria _(M arm (left))_: Metria-IH1 mounted upper left arm; 6MWT: six minute walk test. | | | | | | | |

Table ST7. Games-Howell multiple comparison post-hoc test to assess absolute agreement (EE estimates in stroke group with left impairment).

| Spectrum of activities | Gold standard EE estimate | SPA derived EE estimates from different locations | Mean Difference (I-J) | Std. Error | Sig. | 98.4% Confidence Interval | |
| --- | --- | --- | --- | --- | --- | --- | --- |
|  |  |  |  |  |  | Lower Bound | Upper Bound |
| Lying | Cosmed K4B2 | A _arm (right)_ | 1.58 | 0.16 | .001* | 0.75 | 2.43 |
|  |  | A _waist (right)_ | 1.47 | 0.20 | .000* | 0.66 | 2.28 |
|  |  | A _ankle (right)_ | 1.57 | 0.16 | .001* | 0.74 | 2.40 |
|  |  | Metria _(M arm (left))_ | -0.06 | 0.21 | .998 | -0.90 | 0.78 |
| Sitting | Cosmed K4B2 | A _arm (right)_ | 1.25 | 0.11 | .000* | 0.75 | 1.77 |
|  |  | A _waist (right)_ | 1.25 | 0.11 | .000* | 0.75 | 1.76 |
|  |  | A _ankle (right)_ | 1.28 | 0.10 | .000* | 0.75 | 1.83 |
|  |  | Metria _(M arm (left))_ | -0.33 | 0.16 | .296 | -0.98 | 0.31 |
| Standing | Cosmed K4B2 | A _arm (right)_ | 1.39 | 0.23 | .001* | 0.48 | 2.31 |
|  |  | A _waist (right)_ | 1.54 | 0.17 | .001* | 0.65 | 2.44 |
|  |  | A _ankle (right)_ | 1.53 | 0.17 | .001* | 0.64 | 2.43 |
|  |  | Metria _(M arm (left))_ | -0.16 | 0.22 | .950 | -1.06 | 0.74 |
| 50 steo walk | Cosmed K4B2 | A _arm (right)_ | 0.14 | 0.85 | 1.000 | -3.78 | 4.06 |
|  |  | A _waist (right)_ | 1.11 | 0.94 | .763 | -3.30 | 5.52 |
|  |  | A _ankle (right)_ | -2.96 | 0.98 | .110 | -7.60 | 1.68 |
|  |  | Metria _(M arm (left))_ | 1.01 | 0.39 | .162 | -0.67 | 2.69 |
| 6MWT | Cosmed K4B2 | A _arm (right)_ | -0.30 | 2.36 | 1.000 | -9.91 | 9.31 |
|  |  | A _waist (right)_ | 1.12 | 2.73 | .993 | -10.39 | 12.63 |
|  |  | A _ankle (right)_ | -3.72 | 2.32 | .528 | -13.16 | 5.71 |
|  |  | Metria _(M arm (left))_ | 0.44 | 1.83 | .999 | -7.17 | 8.05 |
| Multi sit-to-stand | Cosmed K4B2 | A _arm (right)_ | -1.61 | 1.59 | .842 | -8.22 | 5.00 |
|  |  | A _waist (right)_ | -0.50 | 1.77 | .998 | -8.11 | 7.10 |
|  |  | A _ankle (right)_ | 4.84 | 0.90 | .015* | 0.06 | 9.63 |
|  |  | Metria _(M arm (left))_ | 2.92 | 0.91 | .104 | -1.76 | 7.61 |

*. The mean difference is significant at the 0.016 level. EE: Energy expenditure; Spa: Standard proprietary algorithm; A _arm (right)_: ActiGraph G3Tx mounted at right arm; A _waist (right)_: ActiGraph G3Tx mounted at right waist; A _ankle (right)_: ActiGraph G3Tx mounted at right ankle; Metria _(M arm (left))_: Metria-IH1 mounted upper left arm; 6MWT: six minute walk test.

Table ST8. Games-Howell multiple comparison post-hoc test to assess absolute agreement (MET estimates in stroke group with left impairment).

| Spectrum of activities | Gold standard MET estimate | SPA derived MET estimates from different locations | Mean Difference (I-J) | Std. Error | Sig. | 98.4% Confidence Interval | |
| --- | --- | --- | --- | --- | --- | --- | --- |
|  |  |  |  |  |  | Lower Bound | Upper Bound |
| Lying | Cosmed K4B2 | A _arm (right)_ | 0.07 | 0.02 | .11 | -0.05 | 0.19 |
|  |  | A _waist (right)_ | 0.05 | 0.03 | .45 | -0.07 | 0.17 |
|  |  | A _ankle (right)_ | 0.07 | 0.02 | .11 | -0.05 | 0.19 |
|  |  | Metria _(M arm (left))_ | -0.06 | 0.08 | .92 | -0.46 | 0.33 |
| Sitting | Cosmed K4B2 | A _arm (right)_ | -0.12 | 0.05 | .24 | -0.37 | 0.14 |
|  |  | A _waist (right)_ | -0.11 | 0.05 | .27 | -0.37 | 0.14 |
|  |  | A _ankle (right)_ | -0.11 | 0.05 | .27 | -0.37 | 0.14 |
|  |  | Metria _(M arm (left))_ | -0.24 | 0.08 | .08 | -0.56 | 0.08 |
| Standing | Cosmed K4B2 | A _arm (right)_ | -0.02 | 0.11 | 1.00 | -0.48 | 0.44 |
|  |  | A _waist (right)_ | 0.04 | 0.09 | .99 | -0.44 | 0.52 |
|  |  | A _ankle (right)_ | 0.04 | 0.09 | .99 | -0.44 | 0.52 |
|  |  | Metria _(M arm (left))_ | -0.12 | 0.09 | .73 | -0.58 | 0.35 |
| 50 steo walk | Cosmed K4B2 | A _arm (right)_ | -0.04 | 0.38 | 1.00 | -1.93 | 1.86 |
|  |  | A _waist (right)_ | 0.28 | 0.38 | .94 | -1.61 | 2.16 |
|  |  | A _ankle (right)_ | -2.18 | 0.29 | .001* | -3.55 | -0.83 |
|  |  | Metria _(M arm (left))_ | 0.65 | 0.13 | .004* | 0.13 | 1.18 |
| 6MWT | Cosmed K4B2 | A _arm (right)_ | -0.35 | 1.00 | 1.00 | -4.60 | 3.90 |
|  |  | A _waist (right)_ | 0.70 | 1.23 | .98 | -4.84 | 6.25 |
|  |  | A _ankle (right)_ | -2.70 | 0.78 | .04 | -5.83 | 0.44 |
|  |  | Metria _(M arm (left))_ | 0.03 | 0.66 | 1.00 | -2.67 | 2.74 |
| Multi sit-to-stand | Cosmed K4B2 | A _arm (right)_ | -1.14 | 0.62 | .41 | -3.68 | 1.41 |
|  |  | A _waist (right)_ | -0.33 | 0.86 | .99 | -4.23 | 3.56 |
|  |  | A _ankle (right)_ | 2.16 | 0.37 | .01* | 0.22 | 4.12 |
|  |  | Metria _(M arm (left))_ | 1.78 | 0.37 | .02* | -0.14 | 3.71 |

*. The mean difference is significant at the 0.016 level. MET: Metabolic equivalent; Spa: Standard proprietary algorithm; A _arm (right)_: ActiGraph G3Tx mounted at right arm; A _waist (right)_: ActiGraph G3Tx mounted at right waist; A _ankle (right)_: ActiGraph G3Tx mounted at right ankle; Metria _(M arm (left))_: Metria-IH1 mounted upper left arm; 6MWT: six minute walk test.
